# Specific Caleosin/Peroxygenase and Lipoxygenase Activities Are Tissue-Differentially Expressed in Date Palm (*Phoenix dactylifera* L.) Seedlings and Are Further Induced Following Exposure to the Toxin 2,3,7,8-tetrachlorodibenzo-p-dioxin

**DOI:** 10.3389/fpls.2016.02025

**Published:** 2017-01-06

**Authors:** Abdulsamie Hanano, Ibrahem Almousally, Mouhnad Shaban, Farzana Rahman, Mehedi Hassan, Denis J. Murphy

**Affiliations:** ^1^Department of Molecular Biology and Biotechnology, Atomic Energy Commission of SyriaDamascus, Syria; ^2^Genomics and Computational Biology Group, University of South WalesWales, UK

**Keywords:** date palm, *Phoenix dactylifera*, caleosins, peroxygenase, lipoxygenase, TCDD, dioxins

## Abstract

Two caleosin/peroxygenase isoforms from date palm, *Phoenix dactylifera* L., PdCLO2 and PdCLO4, were characterized with respect to their tissue expression, subcellular localization, and oxylipin pathway substrate specificities in developing seedlings. Both PdCLO2 and PdCLO4 had peroxygenase activities that peaked at the mid-stage (radicle length of 2.5 cm) of seedling growth and were associated with the lipid droplet (LD) and microsomal fractions. Recombinant PdCLO2 and PdCLO4 proteins heterologously expressed in yeast cells were localized in both LD and microsomal fractions. Each of the purified recombinant proteins exhibited peroxygenase activity but they were catalytically distinct with respect to their specificity and product formation from fatty acid epoxide and hydroxide substrates. We recently showed that date palm CLO genes were upregulated following exposure to the potent toxin, 2,3,7,8-tetrachlorodibenzo-*p*-dioxin (TCDD) (Hanano et al., 2016), and we show here that transcripts of 9- and 13-lipoxygenase (LOX) genes were also induced by TCDD exposure. At the enzyme level, 9-LOX and 13-LOX activities were present in a range of seedling tissues and responded differently to TCDD exposure, as did the 9- and 13-fatty acid hydroperoxide reductase activities. This demonstrates that at least two branches of the oxylipin pathway are involved in responses to the environmental organic toxin, TCDD in date palm.

## Introduction

Caleosins (Pfam reference PF05042) are a group of structurally related genes that appear to be ubiquitous in Streptophytes, including land plants, and in the Chlorophyte green algae; while similar genes are also found in most, but not all, fungal clades (Naested et al., 2000; Partridge and Murphy, 2009; Hanano et al., 2015a). The proteins encoded by caleosin genes share a single highly conserved calcium-binding, EF-hand motif plus an invariant heme-binding histidine residue in the region proximal to the N terminus. This is followed by a relatively hydrophobic, potentially membrane-spanning, region plus a proline rich domain in the center of the protein. Finally, there is a region containing several predicted kinase sites proximal to the C terminus (Khalil et al., 2014; Lizong et al., 2014; Shen et al., 2014; Song et al., 2014; Charuchinda et al., 2015). Experimental studies have confirmed that caleosins from both plants and fungi can act as calcium binding proteins and have a lipid peroxygenase (PXG) activity that depends on the presence of a heme group coordinated by the invariant histidine residue (Hanano et al., 2006, 2015a; Blée et al., 2012). The lipid peroxygenase activity is associated with epoxy fatty acid biosynthesis as part of overall oxylipin metabolism in plants (Hanano et al., 2006; Blée et al., 2012). These caleosin/peroxygenase proteins have been shown to be associated with bilayer membranes and/or lipid droplets (LDs) with some isoforms being able to bind to both types of lipid structure (Naested et al., 2000; Partridge and Murphy, 2009; Purkrtova et al., 2015).

To date, only a relatively small number of the many hundreds of plant and fungal genes currently annotated as “caleosin” and/or “peroxygenase” in public databases, such as NCBI or Ensembl Plant, have been shown to encode proteins with experimentally proven PXG activity. Moreover, manual curation of these annotated genes and their derived protein sequences shows that in some cases they lack critical residues involved in key functions such as calcium binding, heme coordination or membrane attachment. In addition, one of the unusual features of caleosin/peroxygenase proteins is that they are the second most highly abundant components (after oleosins) in the proteome of plant LDs (Murphy, 2001, 2012). Caleosins may therefore play important structural roles in facilitating LD assembly and storage in plant tissues in addition to their catalytic functions (Partridge and Murphy, 2009; Hanano et al., 2015a). Caleosin/peroxygenases have also been implicated in a wide range of physiological functions in plants, including lipid packaging, and mobilization in seeds (Murphy, 2001, 2012; Froissard et al., 2009; Purkrtova et al., 2015), drought responses (Aubert et al., 2010; Sham et al., 2014), disease responses (Sham et al., 2014, 2015) and toxin sequestration (Hanano et al., 2016).

We have recently demonstrated that seed LDs from date palm can act as extremely effective sequestration agents for the highly persistent organic toxin, 2,3,7,8-tetrachlorodibenzo-*p*-dioxin (TCDD) (Hanano et al., 2016). We also found that exposure of date palm seedlings to TCDD resulted in a strong transcriptional induction of some members of the caleosin gene family. Given the multifunctional role of caleosin/peroxygenases, it is possible that these proteins might be involved in the plant response to TCDD at several levels, i.e., in facilitating LD accumulation in order to assist toxin sequestration and also as part of an oxylipin signaling pathway involved in the overall stress response. Another possibility is that the caleosin/peroxygenases might play a role in ameliorating the oxidative stress that is one of the consequences of exposure to dioxins (Bagchi and Stohs, 1993).

Chemically speaking, dioxins are polychlorinated dibenzo-*p*-dioxins (PCDDs) and polychlorinated dibenzofurans (PCDFs); they are structurally stable, extremely hydrophobic compounds that characteristically exhibit high affinities toward lipids. Such characteristics enable dioxins to persist for many years in the environment and to bioaccumulate in food chains. It also means that they constitute the most toxic group of persistent organic pollutants (POPs) that is found in the wider environment, including in soils and marine and freshwater systems, from where they can be taken up by animals. The toxic effects of TCDD are numerous and mostly result from oxidative stress (Bagchi and Stohs, 1993), lipid peroxidation (Stohs, 1990), DNA damage (Wahba et al., 1988), and changes in membrane fluidity (Alsharif et al., 1990). Several mechanisms have been proposed for the toxicity of TCDD (Poland and Knutson, 1982; Pohjanvirta and Tuomisto, 1994), with oxidative stress considered as one of the more important components (Stohs, 1990; Latchoumycandane et al., 2003).

Plants are also exposed to environmental dioxins. These toxins are not used for nutrition or as sources of energy but are taken up from the external environment and tend to accumulate in several plant tissues. For example, a variety of zucchini (*Cucurbita pepo* L.) accumulated various dioxin and dioxin-like congeners in the root system to varying degrees with more hydrophobic types being accumulated to a greater extent (Inui et al., 2011). Likewise, Arabidopsis plants can absorb such xenobiotics from the external environment, accumulating them in leaves, seeds and roots (Hanano et al., 2014a, 2015b). These and other findings have led to the concept of using such plants for extracting, sequestering and detoxifying dioxins from the environment. This “green” process is part of the agenda for bioremediation and has potential for the total mineralisation of organic pollutants (Aken et al., 2010).

However, plants such as zucchini and Arabidopsis are unsuitable for use in all climatic regions. This is especially true for relatively hot, dry, and saline-affected parts of the world including the Middle East and parts of southern Asia where pollution by organic chemicals is an increasing issue (Hanano et al., 2014b). For this reason, we are investigating the relationship between plants native to these regions and environmental xenobiotics in order to identify potential candidates to serve as “clean-up” agents. One such candidate is date palm, which is highly resistant to drought, easy to cultivate over a large area, and has an extensive and efficient root system. In our recently developed method, LDs from date palm seeds were used to extract dioxins from aquatic environments without adversely affecting the young date palm seedlings (Hanano et al., 2016). This contrasts with Arabidopsis plants which when treated by TCDD exhibited decreases in fresh weight, chlorophyll content, seed germination, and increases in levels of hydrogen peroxide (H_2_O_2_) and fatty acid hydroperoxides (FAOOHs) (Hanano et al., 2014a, 2015b). These latter phenotypes resemble to those of *rd20* null mutants that were exposed to oxidative stress conditions. The RD20 (for *R*esponsive to *D*ehydration *20*,) protein is a caleosin isoform in Arabidopsis that is found in subcellular membranes and at the surface of LDs in several plant tissues (Takahashi et al., 2000; Aubert et al., 2010, 2011; Shen et al., 2014, 2016; Hanano et al., 2015c). The RD20 caleosin has also been shown to be a peroxygenase that reduces fatty acid hydroperoxides to their corresponding alcohols (FAOH) and such alcohols have been shown to modulate oxidative stress and cell death, conferring resistance against biotic and abiotic stresses (Blee et al., 2014; Hanano et al., 2015c). The same RD20 caleosin is involved in the regulation of a range of physiological processes including stomatal control, transpiration, drought tolerance, seed germination, and G protein signaling (Aubert et al., 2010, 2011; Ehdaeivand, 2014; Khalil et al., 2014; Wright, 2014).

We recently found that the expression of two of the five caleosin-like genes present in the date palm genome increased strongly following exposure of plants to TCDD, suggesting a possible role of caleosins in the response to xenobiotic exposure (Hanano et al., 2016). In order to investigate more fully the roles of caleosin/peroxygenases in the physiological response of plants to dioxins, and to investigate the feasibility of using such responses as part of plant-based environmental remediation strategies, we have further characterized the properties and regulation of two caleosins, both *in situ* and as recombinant proteins expressed in yeast. We have also investigated several upstream lipoxygenase and reductase activities at transcriptional and biochemical levels with regard to their tissue location, substrate specificity and upregulation following toxin exposure. The implications of the results for understanding plant responses to organic toxins and the potential for biotechnological exploitation of such responses are also discussed.

## Materials and methods

### Plant materials, conditions and TCDD-treatment

Seeds of date palm (*Phoenix dactylifera* L.), variety Khalas were washed, air-dried, and stored in plastic bags at room temperature. Seeds were germinated *in vitro* as described previously (Hanano et al., 2016). Seedlings were obtained 15 days after sowing. Seedlings with a radicle length of 0.5, 2.5, or 5 cm were referred as stage I, II, and III, respectively. Non-germinated seeds (stage 0). The 2,3,7,8-tetrachlorodibenzo-p-dioxin (2,3,7,8-TCDD dissolved in toluene at 10 μg mL^−1^, purity 99%) was purchased from Supelco Inc., USA. To test the effect of TCDD, seeds were daily watered by the prepared solutions at various concentrations of TCDD (0, 10, 50 ng L^−1^) as described previously (Hanano et al., 2016).

### Strains, culture conditions and chemicals

*Escherichia coli* strain TOP10 was used as host for plasmid cloning experiments. Bacteria were grown in Luria–Bertani medium supplemented with ampicillin (100 mg mL^−1^) at 37°C. *Saccharomyces cerevisiae* Wa6 (*ade, his7-2 leu2-3 leu2-112 ura3-52*) the host for protein expression studies. Recombinant yeast was grown in S medium (7 g L^−1^ yeast nitrogen base, 1 g L^−1^ casamino acids, 20 g L^−1^ glucose supplemented with 50 mg mL^−1^ histidine, 200 mg mL^−1^ adenine, and 50 mg mL^−1^ leucine) for 2 days with shaking at 30°C. Culture media components and agarose were purchased from Sigma-Aldrich, USA and liquid-chromatography grade solvents and buffer components were supplied by Merck, Germany. Isopropyl-b-D-thiogalactopyranoside (IPTG), X-Gal, restriction enzymes, RNase A, molecular markers for DNA, and dNTPs were purchased from Fermentas (Vilnius, Lithuania). Oligonucleotides, aniline, thiobenzamide, cumene hydroperoxide, fatty acids, antibiotics, and amino acids were supplied by Sigma-Aldrich, USA.

### Isolation of LDs from date palm seedlings

LDs were isolated from seeds of date palm (*Phoenix dactylifera* L.) according to Hanano et al. (2016) with minor modifications. Seeds of date palm were firstly subjected to dry grinding using a high performance grinder (Cross Beater Mill SK, Retsch, Germany). Well-hulled and crushed grains were obtained and brief sieving was used to separate the woody cover particles of the seeds from their stony cores (0.5 mm). Five grams of the ground core were taken and milled in a brass mortar in the presence of liquid nitrogen until a fine powder was obtained. The powder (5 g) was immediately hydrated with 10 mL of buffer A (100 mM potassium pyrophosphate, 0.1 M sucrose, and pH 7.4). The mixture was gently homogenized for 5 min using an ultra-dispenser (T25 digital ULTRA-TURRAX, IKA laboratory, Germany) and centrifuged for 10 min at 10,000 × g. The resulting supernatant was subjected to a second centrifugation at 100,000 × g for 1 h and a floating white pad, consisting of LDs, was collected from the top of the tube. LDs were washed twice with 5 mL of buffer B (buffer A without sucrose). After a final centrifugation (100,000 × g for 1 h), the LD fraction was suspended in 2 mL of buffer B and stored at 4°C for further analysis.

### Enzyme assays

Peroxygenase activity was measured by the oxygenation of aniline or thiobezamide as substrates (Blee and Durst, 1987). Hydroperoxide-reductase activity was measured by incubation of 9-HPOD or 13-HPOD overnight at 26°C with 50 μg of purified recombinant proteins in 500 μL of sodium acetate (0.1 M, pH 5.5) (Hanano et al., 2015b). Residual substrate and products were extracted in 3 × 2 mL of dichloromethane/ether (1:1, v/v). After drying under nitrogen flow, extracts were taken with 25 μL of acetonitrile/water/acetic acid (50/50/0.1, v/v/v). Extracts were analyzed using a Jasco LC-2000 plus series HPLC system (Jasco, USA) with a UV-detector (RF-10Axl, Shimadzu) (234 nm) and a C18 column (Eclipse XDB-C18 150 × 4.6 mm, 5 μm; Agilent, USA). The analysis was performed using a mobile phase of acetonitrile/water/acetic acid (50/50/0.1, v/v/v) at a flow rate of 0.6 mL min^−1^.

For analysis of lipoxygenase (LOX) activities in date palm seedling tissues, two grams of fresh tissues (plumule, petiole, or radicle) were ground into a fine powder in liquid nitrogen with a mortar and a pestle, followed by resuspension in 100 μL of enzyme extraction buffer (50 mM sodium phosphate buffer, pH 7.5, 10 mM EDTA, 0.1% Triton X-100). The homogenate was centrifuged at 4°C, 16,000 × g for 10 min to remove cell debris. Total protein content was determined by a Bradford assay. LOX activity was determined according to Hanano et al. (2015b) in 500 μl of 50 mM sodium phosphate buffer (pH 7.0) containing 50 μg of proteins extract and 500 μM of linoleic acid (C18:2) at 25°C with constant shaking for 30 min. The reaction products were extracted in 5 mL *n*-hexane/2-propanol (3/2) (v/v) with 0.025% (w/v) butylated hydroxytoluene and the mixture immediately ultra-homogenized for 30 s on ice. A spiked sample with 100 mM of each hydroperoxide was used as a control. The extract was shaken for 10 min and centrifuged at 3000 × g at 4°C for 10 min. The upper phase was then dried under nitrogen. Hydroperoxides were dissolved in 25 μl acetonitrile/water/acetic acid (50/50/0.1) (v/v/v) and quantified by HPLC as described above. The analysis was performed using a mobile phase of acetonitrile/water/acetic acid (50/50/0.1, v/v/v) at a flow rate of 0.6 mL min^−1^. FA-hydroperoxides were quantified using their respective standards. One unit of activity (U) corresponds to the amount of enzyme that converts 1 μmol of substrate per minute.

### Primer design and PCR amplification conditions

Nucleotide sequences of primers used in this study are shown in Table S1. The PCR amplification was performed in a 25 μl reaction final volume containing 3 mM MgSO_4_, 200 μM each of the four dNTPs, 10 μM of each primers and 2.5 U *Taq* DNA polymerase. PCR conditions were: 94°C for 4 min followed by 35 cycles at 94°C for 30 s, 58°C for 30 s, 72°C for 1 min and 72°C for 10 min. PCR amplifications were verified by 1% agarose gel electrophoresis for 30 min at 100 V. The gel was visualized in an UV illuminator. If necessary, PCR fragments were purified using a QIAquick PCR purification kit (Qiagen, USA).

### Cloning and sequence analysis of *PdCLO* genes

Full-length ORFs of *PdCLO2* and *PdCLO4* genes (720 and 711 bp, respectively) were amplified by PCR using date palm cDNA. For further purification of recombinant proteins, primers were designed to add a His tag at the N-terminal ends of the *PdCLO2* and *PdCLO4* genes (Table S1). Purified *PdCLO2* and *PdCLO4-*PCR products were ligated to pGEM-T Easy vector (Promega, USA) following the manufacturer's manual. The recombinant plasmids pGEM-T/*PdCLO2* and pGEM-T/*PdCLO4* were transferred into *E. coli* Top10 competent cells. Recombinant plasmids were extracted from *E. coli* Top10 using a plasmid purification mini kit (Qiagen, Germany). The presence of *PdCLO2* and *PdCLO4* genes in the isolated plasmid was confirmed by PCR and *SacI-EcoRI* digestion. At least three clones of both genes were sequenced on both strands using T7 and SP6 primers on an ABI 310 Genetic Analyser (Applied Biosystems) using Big Dye Terminator kit (Applied Biosystems). Nucleotide sequence analysis and multiple alignments of the nucleotide and deduced amino acid sequences were performed with Vector NTI advance (Invitrogen).

### Expression of recombinant proteins

*PdCLO2* and *PdCLO4* were subcloned into the yeast constitutive expression vector pVT102U (Vernet et al., 1987) using *SacI* and *EcoRI* restriction sites. The recombinant plasmids pVT102U*/PdCLO2* and pVT102U*/PdCLO4* were then introduced into the yeast *Saccharomyces cerevisiae* Wa6 (*ade, his7-2 leu2-3 leu2-112 ura3-52*) (Schiestl and Gietz, 1989). Expression of the recombinant proteins in transformed yeast cells was carried out as described previously (Hanano et al., 2006). The LD and microsomal fractions of recombinant yeast cells were extracted and resuspended in 10 mM potassium phosphate (pH 8) containing 10% glycerol (v/v) and treated with the non-polar detergent, emulphogene, (final concentration, 0.2%) for 45 min at 4°C. The mixture was then centrifuged at 100,000 × g for 2 h to removed non-solubilised material.

### Purification of recombinant proteins, SDS-PAGE and western blotting

The LDs of recombinant yeast cultures resuspended in 10 mM potassium phosphate (pH 8) containing 10% glycerol (v/v) were treated separately with emulphogene (Sigma-Aldrich, USA) at final concentration of 0.2% (v/v) for 45 min. The mixture was then centrifuged at 100,000 × g for 45 min. The supernatant was taken and the concentration of solubilized proteins was estimated by Bradford assay and enzymatic activity. The solubilized fraction was used for protein purification. All steps were performed on ice (4°C). His-tagged PdCLO2 and PdCLO4 from in the supernatant were purified on a Ni-NTA Superflow column (Qiagen) under native (non-denaturing) conditions, according to the manufacturer's instructions. The purity of the recombinant proteins was confirmed by SDS-12% PAGE followed by Coomassie-Blue staining. For western blot experiment, proteins were fractionated by 12% SDS-PAGE and electroblotted to Immobilon-P membranes (Millipore Corp., Bedford, MA) using a mini-transblot transfer cell apparatus (Bio-Rad). For detection of the recombinant CLO:His fusion protein, a mouse monoclonal anti-His antibody and an anti-mouse antibody conjugated to peroxidase were used at 1:2000. Blots were developed using the ECL kit from Pierce.

### Quantification of gene transcripts

The changes in relative transcriptional abundance of genes encoding *PdCLO2, PdCLO4, 9-LOXs,* and *13-LOXs* in response to TCDD exposure were analyzed by reverse-transcription quantitative PCR (RT-qPCR). Briefly, frozen fine powder (1 g) samples from whole seedlings at stages 0, I, and II or specific tissues were used to extract total RNA using an RNeasy kit according to the manufacturer's instructions (Qiagen, Germany). After quality control, aliquots of 1 μg total RNA were used for first-strand cDNA synthesis according to Hanano et al. (2014a). Real-time PCR was performed in 48-well plates using a AriaMx Real-time PCR System from Agillent technologies, USA. In brief, 25 μL reaction mixtures contained 0.5 μM of each specific oligonucleotide primer for the target genes (*PdCLO2, PdCLO4, 9-LOXs,* and *13-LOXs*) and the reference gene (*TIP-41*) (Table S1), 12.5 μL of SYBR Green PCR mix (Bio-Rad, USA) and 100 ng cDNA. Subsequently, the relative quantification RQ of target genes was calculated directly using the software installed in the qPCR system. The sequences of amplified regions were confirmed by sequencing on an ABI 310 Genetic Analyzer (Applied Biosystems) using a Big Dye Terminator kit (Applied Biosystems).

### Bioinformatics analyses

For sequence retrieval and identifying LOX proteins, eight CLO sequences of *Arabidopsis thaliana* were obtained from Arabidopsis information resources TAIR database (https://www.arabidopsis.org/, release 10) (Berardini et al., 2015). CLO sequences of *Phoenix dactylifera, Elaeis guineensis,* and *Musa acuminate* were retrieved from NCBI (http://www.ncbi.nlm.nih.gov/) (see Table 1 for accession numbers). Five CLO proteins have been identified in the *Phoenix dactylifera* and eight CLO proteins in the *Arabidopsis thaliana* genomes (Shen et al., 2014; Hanano et al., 2016). In this study, six CLO proteins in *Elaeis guineensis* and two CLO proteins in *Musa acuminate* were also identified. Using Arabidopsis LOXs as query sequences, LOX sequences were obtained for *Phoenix dactylifera, Elaeis guineensis, Musa acuminate* from NCBI via local BLAST+ search (Chen et al., 2015). These candidate LOX sequences were analyzed using the InterProScan program (http://www.ebi.ac.uk/interpro/) to confirm the presence of the major LOX domain and PLAT/LH2 (polycystin-1, lipoxygenase, lipoxygenase homology) domain (Jones et al., 2014). CLO and LOX sequences were visually inspected to confirm the presence of LOX and CLO domains.

**Table 1:** **Sequence analysis of caleosin-like proteins in date palm and other selected plants**.

**Species Name**	**Caleosins**	**NCBI Accession**	**Amino acids**	**MW (kDa)**	**Isoelectric point (pl)**	**Fe/Binding^a^**	**Ca**^**2+**^ **Binding- EF hand motif**^b^	
							**Start position**	**End position**
*Phoenix dactilyfera*	PdCLO1	XP_008803896	202	22.27	8.36	H^31^–H^99^	21	57
	PdCLO2	XP_008775946	239	26.49	8.35	H^68^–H^136^	58	94
	PdCLO3	XP_008801250	236	26.6	5.61	H^9^–H^75^	1	33
	PdCLO4	XP_008775947	175	19.26	5.95	H^65^–H^133^	55	91
	PdCLO5	XP_008796441	103	11.92	5.14	H^7^ Only	1	27
*Elaeis guineensis*	EgCLO1	XP_010920301	236	27.15	5.98	H^65^–H^133^	55	91
	EgCLO2	XP_010932570	237	26.21	9.46	H^63^–H^131^	53	89
	EgCLO3	XP_010909571	205	22.73	5.91	H^32^–H^100^	22	58
	EgCLO4	XP_010917508	173	19.89	6.6	H^26^–H^70^	16	49
	EgCLO5	XP_010917507	204	22.81	6.21	H^36^–H^104^	26	62
	EgCLO6	XP_010917509	206	22.5	6.3	H^38^–H^106^	28	64
*Musa acuminata*	MaCLO1	XP_009412834	236	27.15	5.92	H^65^–H^133^	55	91
	MaCLO2	XP_009419119	195	21.69	9014	H^24^–H^92^	14	50
*Arabidopsis thaliana*	AtCLO1	NP_194404	245	28.03	5.81	H^70^–H^138^	60	96
	AtCLO2	AED96604	243	27.87	5.62	H^69^–H^137^	59	96
	AtCLO3	NP_180896	236	26.6	5.17	H^65^–H^136^	55	91
	AtCLO4	NP_564995	195	22.09	9.39	H^22^–H^90^	58	94
	AtCLO5	AEE30361	210	23.85	9.62	H^33^–H^101^	23	59
	AtCLO6	NP_564996	192	21.51	9.04	H^23^–H^91^	13	49
	AtCLO7	NP_173739	205	23.8	9.77	None	21	57
	AtCLO8	AED93866	220	24.85	8.78	H^68^–H^138^	58	94

*Accession numbers were parsed from NCBI database (http://www.ncbi.nlm.nih.gov). Molecular weights and isoelectric points were calculated using ExPASy*.

a *Position of histidine residues involved in iron binding (heme axial ligand)*.

b *Position of Ca^2+^Binding- EF hand motif residues*.

In this study, we identified 34 LOX proteins among the four analyzed species and divided them into two groups: 9-LOX and 13-LOX. This division is supported by the phylogenetic tree analysis as discussed below. Net charge for each LOX protein was calculated using BACHEM (http://www.bachem.com/service-support/peptide-calculator/). Physical and chemical properties of LOX and CLO proteins (Molecular Weight (MW), isoelectric point (pI), amino acid length) were computed using ExPASY (http://www.expasy.org/protscale/) (Gasteiger et al., 2003).

Multiple sequence alignment and domain analysis were performed using ClustalOmega software version 1.2.2 for 9- LOX, 13-LOX, and CLO sequences using default parameters (Sievers et al., 2011). The alignments were inspected using CLC sequence viewer (Figures S1, S2) (http://www.clcbio.com). Complete alignments with RasMol color codes (Sayle and Milner-White, 1995), are available in the supplementary documents sections. The amino acid sequence alignments were used to construct a phylogenetic tree using ClustalW2 program version 2.1. The tree was generated following Bayesian Inference (BI), Neighbour-joining (NJ), and Unweighted Pair Group Method with Arithmetic Mean (UPGMA) methods. The tree topologies constructed using the three methods showed complete consistency. The NJ tree was constructed using ClustalW2 program version 2.1 (Larkin et al., 2007). The constructed tree was inspected using FigTree and is shown in Figure 8 (Rambaut and Drummond, 2009). Multiple sequence alignment of caleosin sequences from all four species revealed that the Ca^2+^ binding and EF hand motifs were well conserved, except for the single case of AtCLO7. This may indicate AtCLO7 has lost the ability to bind calcium and may explain its very low extent of expression according to transcriptome analysis (Shen et al., 2014, Figure 2). The CLO central hydrophobic domain and proline knot motifs were identified as present in all sequences except PdCLO5 (Figure 2). MEME software was used to investigate whether the 9-LOX and 13-LOX proteins contain the histidine rich motif (Figures S3, S4, Bailey and Elkan, 1994). The overall workflow of the computational methods is shown in Figure S5.

### Statistical analysis

All data presented were expressed as means ± standard deviation (SD). Statistical analysis was performed using STATISTICA software, version10 (StatSoft Inc.). Comparisons between control and treatments were evaluated by ANOVA analysis and multiple comparisons using Duncan's multiple range test.

## Results

### Date palm seedlings exhibit a TCDD-inducible peroxygenase activity

**Figure 1:**
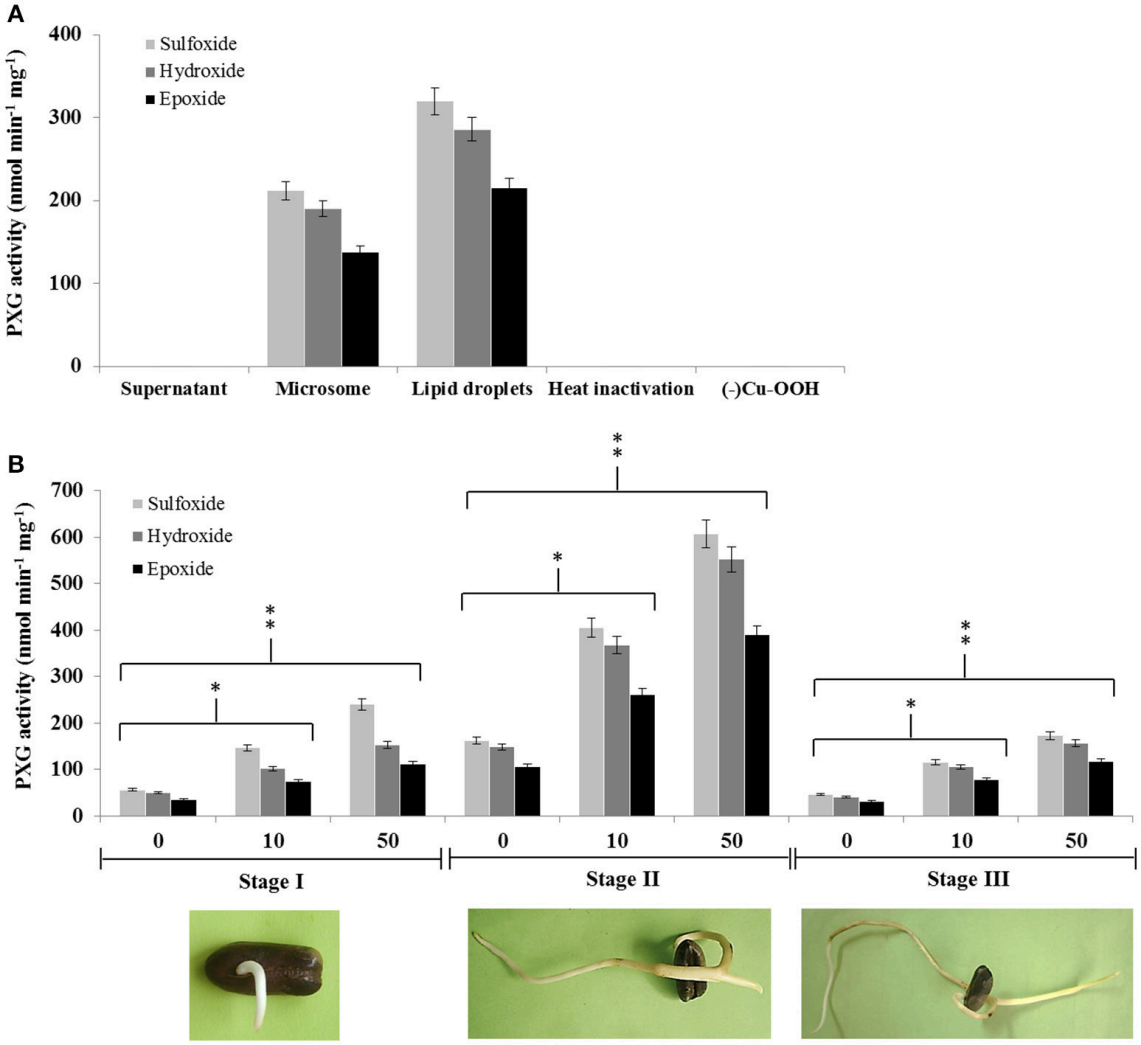
**Peroxygenase activity is associated with LDs and microsomes in date palm seedlings and increases following exposure to TCDD. (A)** PXG activities, sulfoxidation of thiobenzamide (light-gray columns), hydroxylation of aniline (medium-gray columns) and epoxidation of [1-^14^C] oleic acid (dark-gray columns), were measured in supernatant, microsome, and LD fractions from stage II date palm seedlings. **(B)** PXG activities were measured in total crude extracts of seedlings from stages I, II, and III following addition of TCDD at 0, 10, and 50 ng L^−1^. Data are averaged from three independent experiments. Statistical significance was evaluated by ANOVA. Asterisks indicate significant differences between treatment and control: ^*^P < 0.05 (significant); ^**^P < 0.01 (very significant).

Caleosin/peroxygenases (CLO/PXGs) have been reported to be lipid-associated proteins in several plant species. We therefore tested the subcellular distribution of these proteins in date palm seedlings where we found that both microsomes and lipid droplets (LDs) were able to catalyze the same co-oxidation reactions reported for other plant PXGs as follows. Both subcellular fractions oxidized thiobenzamide to its corresponding sulfoxide (212 nmol min^−1^ mg^−1^ of protein in microsomes vs. 320 in LDs), hydroxylated aniline to nitrobezene (190 in microsomes versus 286 in LDs) and epoxidised oleic acid into 9,10-epoxy-stearate (138 in microsomes vs. 216 in LDs), as shown in Figure 1A. In contrast, the supernatant fraction (at 100,000 × g) was unable to catalyze such reactions. Control experiments showed that both microsomes and LDs were inactivated after heat treatment or in absence of the oxygen donor, cumene hydroperoxide (Figure 1A).

We recently reported that the expression of the CLO-encoding genes, *CLO-like2* and *CLO-like4*, was upregulated when seeds were germinated in the presence of TCDD (Hanano et al., 2016). We therefore examined whether PXG activity was induced by TCDD at three different stages of seedling development (I, II, and III). Non TCCD-treated seedlings showed a basal PXG activity in stage I that increased about 3-fold at stage II before declining to its lower level at stage III (Figure 1B). In contrast, TCDD-treated seedlings showed a significant increase in the PXG activity (about 4- to 2.5-fold) in crude extracts from seedlings exposed to 10 or 50 ng L^−1^ TCDD respectively, compared with controls at all three stages of germination (Figure 1B). The highest induction of the PXG activity by TCDD was detected in stage II. Interestingly, no activity was detected in non-germinated seeds at stage 0 (data not shown).

### Sequence analysis of caleosin-like genes and proteins in date palm and other selected plants

**Figure 2:**
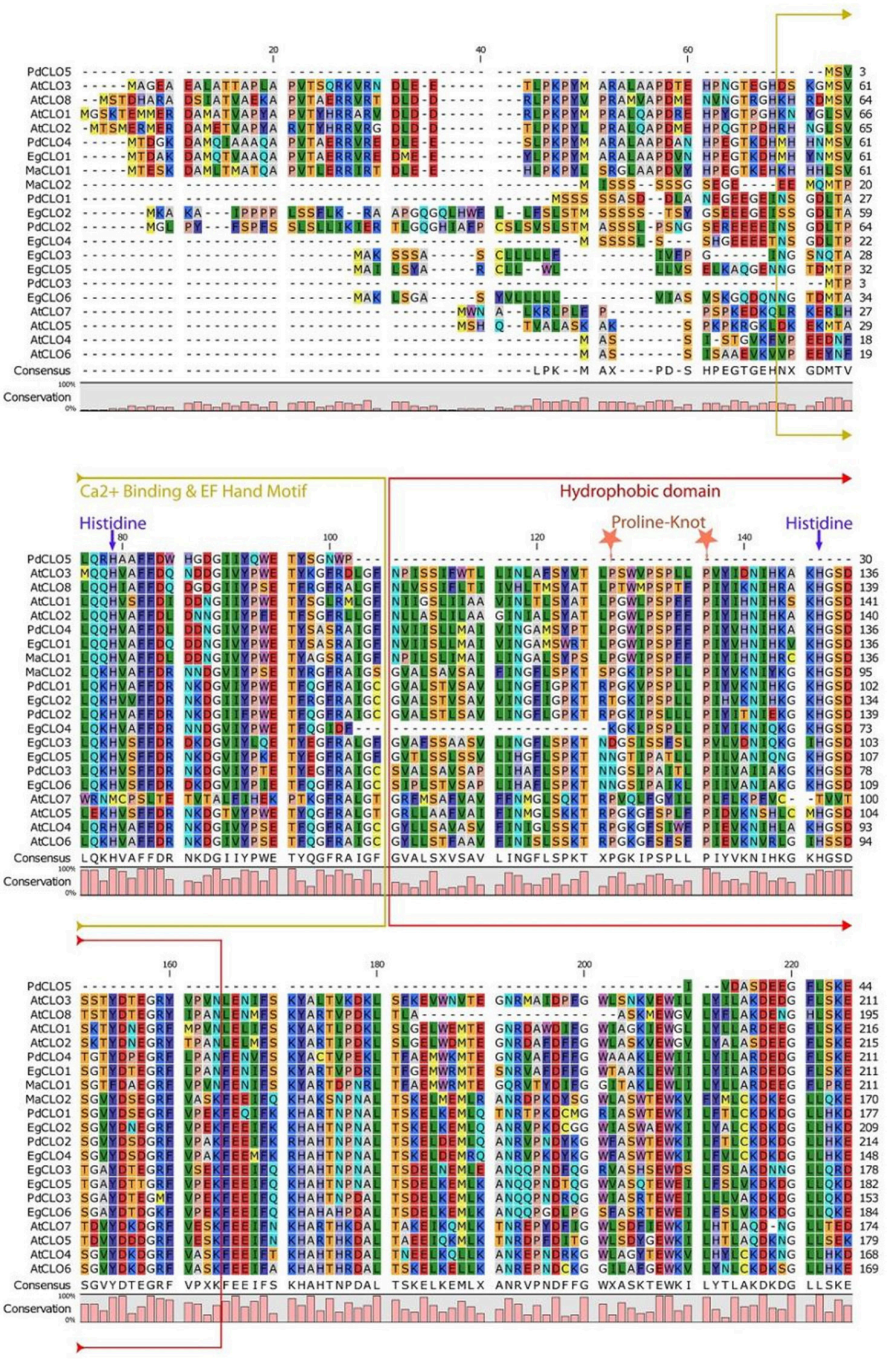
**Amino acid multiple sequence alignment of caleosins from ***Phoenix dactylifera*** (PdCLO1 to PdCLO5), ***Elaeis guineensis*** (EgCLO1 to EgCLO6), ***Musa acuminate*** (MaCLO1 and MaCLO2), and ***Arabidopsis thaliana*** (AtCLO1 to AtCLO8)**. Multiple alignments of caleosin sequences were performed using Clustal Omega (clustalo) from ebi.ac.uk. CLC sequence viewer from CLCBio was used to visualize the alignments. Caleosins from *Phoenix dactylifera, Elaeis guineensis, Musa acuminate,* and *Arabidopsis thaliana* were obtained from the NCBI database (http://www.ncbi.nlm.nih.gov) under accession numbers presented in Table 1. Domains properties of the proteins were analyzed using UniProt platform (http://www.uniprot.org). Color coding is as follows: the multiple sequence alignment residues are colored according to Rasmol amino color scheme, Mustard color boxed residues indicate the combined Ca^2+^ binding EF hand motif, red color boxed residues denote the hydrophobic and putative membrane or lipid associated domain. The invariant heme-binding histidine motif is marked in blue with down arrows (note that the AtCLO7 sequence lacks both of these histidines and, as discussed in the main text this might be a pseudogene). Light orange colored stars indicate the location of the proline knot motif with a length of 10 residues.

The properties and amino acid sequence alignment of predicted CLO-like proteins from date palm, oil palm, banana, and Arabidopsis are shown respectively in Table 1 and Figure 2. We previously showed that the genome of date palm contains five potential orthologous CLO-like sequences (Hanano et al., 2016). However, as noted in Figure 2, the predicted sequence of the PdCLO5 protein was significantly truncated compared to the other caleosins and several canonical caleosin domains were missing. Therefore, although the *PdCLO5* gene was expressed in palm seedlings (albeit at low levels) it seems unlikely that this ortholog has the capacity for the range of enzymatic functions of previously described CLO/PXGs (Hanano et al., 2016). In contrast, the remaining four members of the date palm CLO gene family contained all four typical structural features of previously described plant caleosins, namely; a EF-hand motif containing 12 residues involved in Ca^2+^ binding, a central hydrophobic domain including a proline knot (PXXXPSPXXP), two histidine residues responsible for iron binding (heme axial ligand) and a serine kinase phosphorylation site near the C-terminal (Figure 2).

The accession numbers, number of amino acid residues, molecular weight, position of histidine residues responsible for iron binding, position of calcium binding residues and positions of EF-hand motif of cloned date palm caleosins and other plant caleosins are summarized in Table 1. Although all of these CLO genes were expressed in date palm seedlings (Hanano et al., 2016), only two of them, namely *PdCLO2* and *PdCLO4*, were expressed at high levels and the same two genes were significantly induced following exposure to the dioxin TCDD. To gain more detailed information about the possible involvements of these CLO isoforms, we studied their spatiotemporal expression and enzymatic activities at various stages of post-germinative seedling growth and development.

### Spatial and temporal expression of *PdCLO2* and *PdCLO4* during seed germination

**Figure 3:**
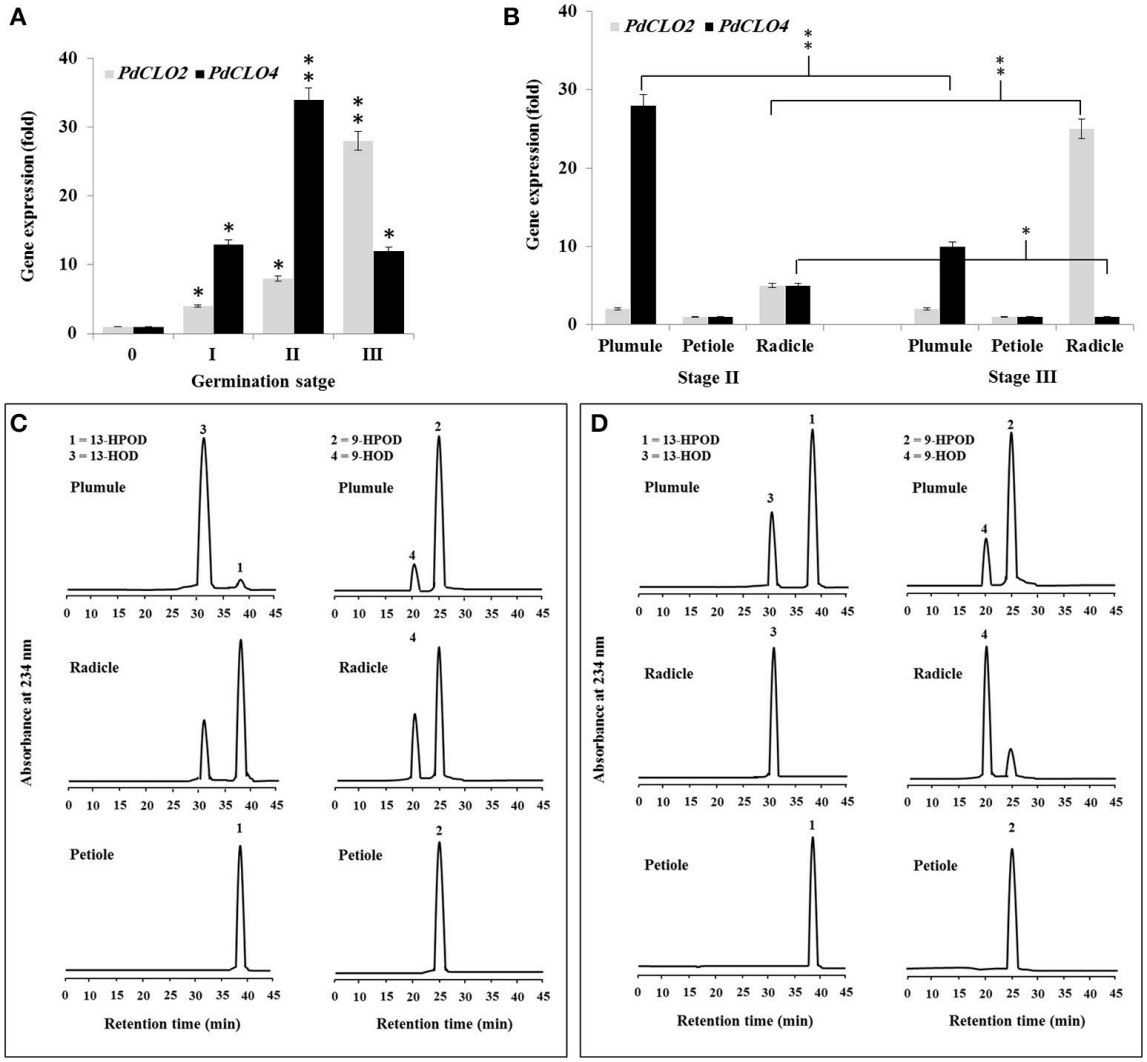
**Transcript abundance and enzymatic activity of PdPXG2 and PdPXG4 in various seedling tissues and developmental stages. (A)** *PdCLO2* and *PdCLO4* transcriptional level analyzed by RT-PCR at different stages of seedling development, 0, I, II, and III. **(B)** *PdCLO2* and *PdCLO4* expression plumule, petiole and radicle tissues for stages II and III. **(C,D)** UV-HPLC analysis of the 13- and 9-HPOD-reductase activities by brut extract of plumule, petiole and radicle from seedling in stage II and II, respectively. Top panels: Products formed after 2 h of incubation, at 27°C, of 13-HPOD or 9-HPOD with plumule extract from stages II and III, respectively. Peak 1 and 2: residual 13-HPOD or 9-HPOD, respectively; peak 3 and 4: 13-HOD or 9-HOD, respectively. Middle panels: Transformation of 13-HPOD or 9-HPOD under the action of radicle extract from stages II and III, respectively. Lower panels: Transformation of 13-HPOD or 9-HPOD under the action of petiole extract from stages II and III, respectively. All measurements were done in triplicate. Values are the means ± S.D. (*n* = 3). Asterisks indicate significant differences in genes expression between different stages of development and between different tissues in stage II and III: ^*^P < 0.05 (significant); ^**^P < 0.01(very significant).

Since PXG activity varied after seed germination (Figure 1B), we used qRT-PCR to assess whether this was due to differential expression of *PdCLO2* or *PdCLO4* genes after seed germination at stages 0, I, II, and III. Transcription of both *PdCLO2* and *PdCLO4* was undetectable at stage 0 but was activated by stage I. *PdCLO4* transcript levels were highly elevated at stage II compared with those of *PdCLO2*. After this, there was a net decline in *PdCLO4* transcripts, while *PdCLO2* transcripts were maximal at stage III (Figure 3A). The increase in *PdCLO4* and *PdCLO2* transcripts in stage II and III, respectively, led us to investigate whether this was related to a specific tissue in the seedling. When transcripts of both genes were evaluated in the plumule, petiole and radicle tissues in stages II and III, the levels of *PdCLO4* gene transcripts were approximately 5.6-fold higher in plumule than in radicle. In contrast, *PdCLO2* was 12-fold more expressed in radicle tissues than in plumule in stage III (Figure 3B). Very small levels of transcripts for both genes were found in petioles.

Variation in *PdCLO4* and *PdCLO2* gene expression in seedling tissues was confirmed by measuring the corresponding enzymatic activities. The 13- and 9-HPOD reductase activity was estimated in crude extracts prepared from plumule, radicle, and petiole in stage I and II respectively. As Figure 3C shows, the maximal 13-HPOD reduction activity was measured in the plumule extract at stage II, where almost all of this hydroperoxide was reduced into 13-HOD (peaks 1 and 2 respectively, panel left-above). In contrast, about 75% of 13-HPOD was transformed into 13-HOD after incubation with radicle extracts (panel left-middle) and no activity was detected in petiole extract (panel left-down). In parallel, 9-HPOD was slightly reduced with plumule extracts but more so with radicle extracts compared with petiole extract (panels right-above middle and down, respectively). An inverse result was observed in stage III where the activity of 13-HPOD was much lower in plumule and was undetectable in radicle and petiole extracts (Figure 3D, panel on left). A brief, a high and a null 9-HPOD-reductase activity was measured in plumule, radicle, and petiole extracts (Figure 3D, panel in right). Together these results indicate that expression of *PdCLO4* and its 13-HPOD-reductase activity is higher in less differentiated leaf tissues while expression of *PdCLO2* and its 9-HPOD-reductase activity was higher in roots. Due to the interesting variation in tissue expression and catalytic activity these two CLO genes, we selected both of them for cloning and expression in a heterologous yeast cell system.

### The recombinant PdCLO2 and PdCLO4 proteins are catalytically distinct

**Figure 4:**
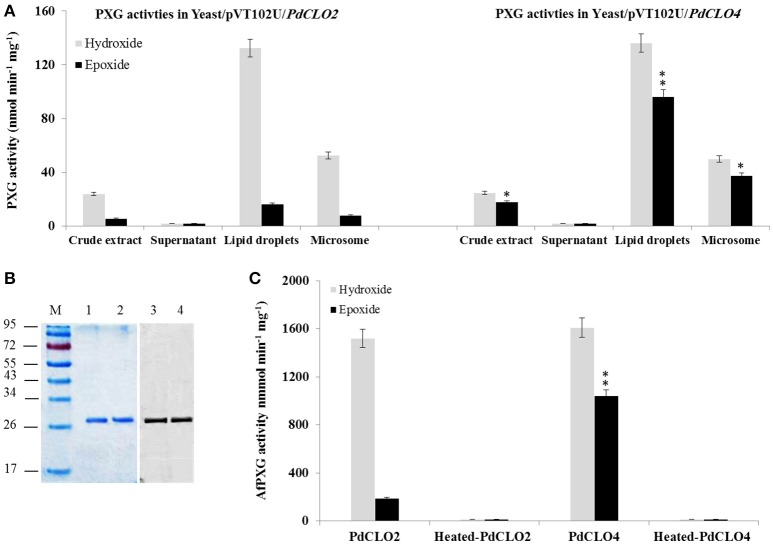
**Expression, purification and specific activity of recombinants PdCLO2 and PdCLO4. (A)** Peroxygenase activities, hydroxylation of aniline (light-green columns) and epoxidation of [1-^14^C] oleic acid (dark-green columns), were measured in the crude extract, supernatant, LD, and microsome fractions of recombinant yeast cultures containing the plasmid pVT102U ligated either with the insert *PdCLO2* or with *PdCLO4*. **(B)** SDS–PAGE analysis of purified PdCLO2 and PdCLO4 (left) and western blotting of the purified proteins with an anti-His antibody (right). **(C)** Specific activities of the purified recombinant PdCLO2 and PdCLO4. Presented values are the means ± S.D. (*n* = 3). ^*^P < 0.05 (significant), ^**^P < 0.01 (very significant).

Following the bioinformatic analysis described above, sequences encoding the PdCLO2 and PdCLO4 isoforms were selected for cloning and separate heterologous expression in yeast in order to test for PXG activity and substrate specificity. The predicted proteins have calculated molecular masses of 26618 and 26732 Daltons respectively. Crude extracts of yeast expressing *PdCLO2* and *PdCLO4* genes efficiently catalyzed hydroxylation of aniline (132.6 vs. 136.2 nmol min^−1^ mg^−1^) and epoxidation of oleic acid (16.2 vs. 96.4 nmol min^−1^ mg^−1^) in the presence of cumene hydroperoxide (Figure 4A). No enzymatic activity was observed in the fractions isolated from yeast transformed with an empty vector (data not shown). When yeast crude extracts were sub-fractionated by differential centrifugation, only microsomes and LDs contained catalytic activity and the LDs from two recombinant yeast strains were more active than their microsomes (Figure 4A). However, the LDs isolated from yeast/pVT102U/*CLO2* were approximately 6-fold less active in epoxidation reactions than LDs isolated from yeast/pVT102U/*CLO4*, while the hydroxylation activity in both was similar.

To confirm and further characterize the enzymatic activity related to PdCLO2 and PdCLO4, we purified each His-tagged isoform separately. LD proteins were solubilized (about 65%) by 0.2% emulphogene and purified by affinity chromatography on a Ni^2+^ column with a purification factor of 11.3 to 11.9 (Table 2). When analyzed by SDS-PAGE, the purified fraction for each protein showed one major band at 26.5 kDa with a degree of purity of >98% as estimated by scanning densitometry and western blotting of the purified proteins with an anti-His antibody confirm their fusion with poly-His tail (Figure 4B). The purified recombinant PdCLO2 and PdCLO4 proteins catalyzed the oxidation of aniline (1.52 versus 1.61 μmol min^−1^ mg^−1^ of protein, respectively) and oleic acid (0.2 vs. 1.05 μmol min^−1^ mg^−1^ of protein, respectively) in the presence of cumene hydroperoxide as co-substrate. Heat-inactivated fractions were unable to catalyze such reactions (Figure 4C). These data strongly suggest that peroxygenase activity in the date palm tissues analyzed is associated with LDs, and to a slightly lesser extent with microsomal membranes.

**Table 2:** **Representative purification of recombinant proteins**.

**Fraction**	**Volume (ml)**	**Total activity (nmol.min^−1^)**	**Protein (mg)**	**(%)**	**Specific activity (nmol.min^−1^.mg^−1^)**	**Purif. factor**
Lipid droplets	4	865.8	6.4	100	135.2	1
Solubilisation	8	1351.4	4.2	65.6	321.7	2.4
Purified CLO-like2	2	3816.8	2.5	39.1	1526.7	11.3
Purified CLO-like4	2	3880.2	2.4	37.5	1616.7	11.9

*Recombinant proteins PdCLO2 and PdCLO4 were solubilized in the presence 0.2% w/v of the detergent emulphogene for 45 min at 4°C. His-tagged PdCLO2 and PdCLO4 were purified by affinity chromatography on a Ni^2+^ column. The activity was measured with 1 mM aniline in the presence of 1 mM cumene hydroperoxide at 310 nm. See text for more details*.

### Fatty acid hydroperoxide specificity of recombinant PdCLO2 and PdCLO4 enzymes

**Figure 5:**
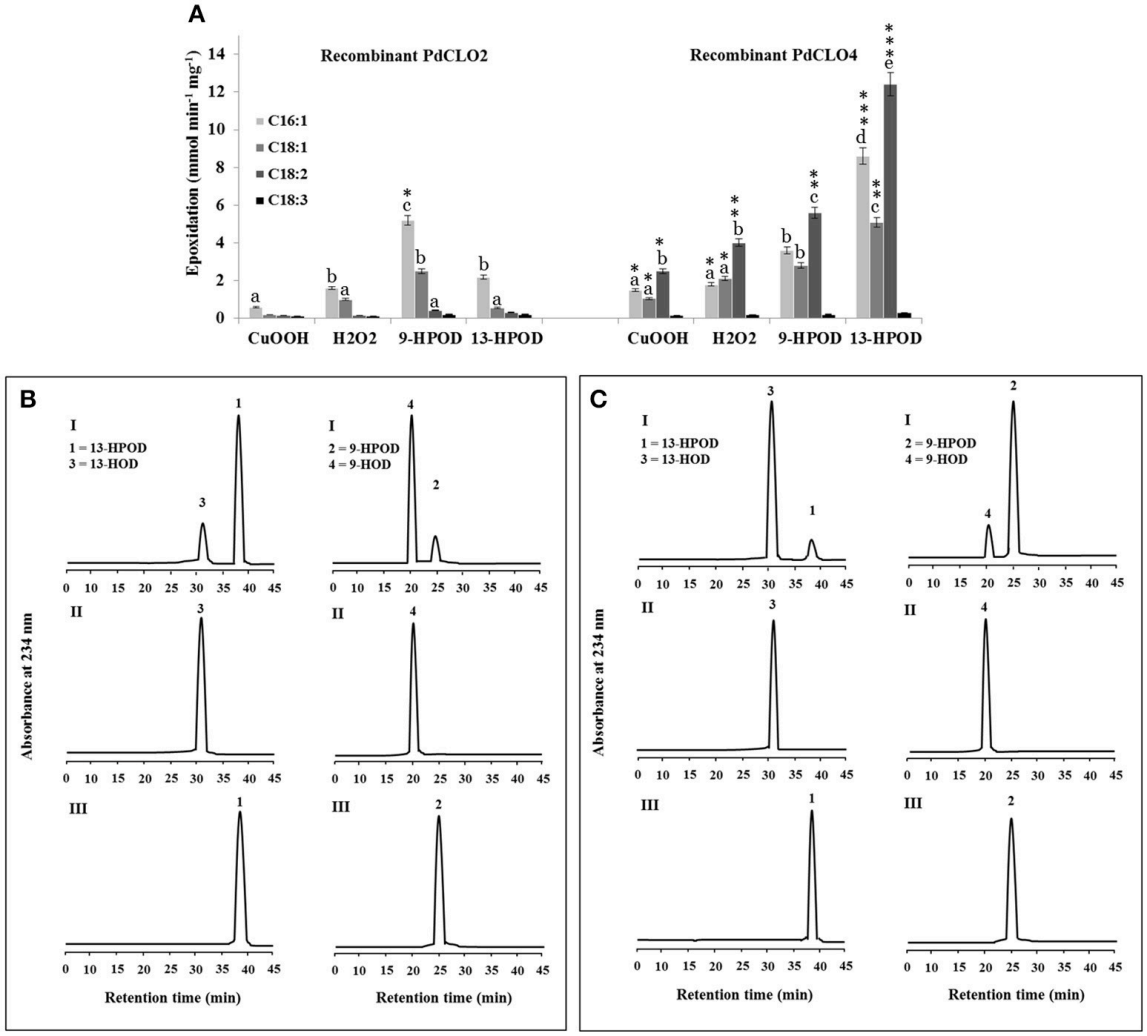
**Fatty acid epoxidation and hydroperoxide metabolism by PdCLO2 or PdCLO4. (A)** Co-oxidation of radiolabelled polyunsaturated fatty acids in the presence of cumene hydroperoxide (CuOOH), hydrogen peroxide (H_2_O_2_), 9-hydroperoxy-10,12-octadecadienoic acid (9-HPOD), or 13-hydroperoxy-9,11-octadecadienoic acid (13HPOD). [^14^C]Substrates metabolized by a purified recombinant PdCLO2 or PdCLO4 were separated by TLC and analyzed by radio-detection. 18:1: oleic acid; 18:2: linoleic acid; 18:3: linolenic acid. **(B,C)** UV-HPLC analysis of the metabolism of fatty acid hydroperoxides by purified PdCLO2 or PdCLO4, respectively. Panel I: Products formed after 2 h of incubation at 27°C of 13-HPOD or 9-HPOD by PdCLO2 and PdCLO4, respectively. Peak 1 and 2: residual 13-HPOD or 9-HPOD, respectively; peak 3 and 4: 13-HOD or 9-HOD, respectively. Panel II: 13-HOD or 9-HOD standards. Panel III: 13-HPOD or 13-HPOD incubated with heat-inactivated PdPXG2 or PdPXG4. Different lowercase letters indicate significant differences (*P* < 0.05) between various fatty acids for each enzyme isoform. Asterisks indicate significant differences between isoforms for each fatty acid (^*^P < 0.05; ^**^P < 0.01; ^***^P < 0.001).

PdCLO2 and PdCLO4 catalyzed epoxidation of oleic acid, a monounsaturated fatty acid (C18:1) at different rates (Figure 4C). Although the majority of plant oxylipins derive from linoleic and linolenic acids (C18:2 and C18:3) (Calvo et al., 1999), these fatty acids were only present in small amounts in date palm seeds (< 10%) while oleic acid (C18:1) constituted about 45% of the total fatty acid fraction (Basuny and Al-Marzooq, 2011). Therefore, the ability of PdCLO2 and PdCLO4 to epoxide mono- and/or polyunsaturated fatty acids was evaluated. [^14^C]-labeled palmitic acid (C16:1), oleic acid (C18:1), linoleic acid (C18:2), or linolenic acid (C18:3) were individually incubated with recombinant PdCLO2 and PdCLO4 in the presence of cumene hydroperoxide, H_2_O_2_ or 9-HPOD and 13-HPOD and their further metabolism followed by radio-TLC. Regardless of the hydroperoxide used, PdCLO2 preferentially epoxidised C16:1 and C18:1 but had much lower activities with C18:2 and C18:3. Conversely, C18:2 > C16:1 > C18:1 were epoxidised by PdCLO4. The most efficient co-substrates for PdCLO2 were 9-HPOD followed by 13-HPOD, H_2_O_2,_ and cumene hydroperoxide. In contrast, PdCLO4 preferred 13-HPOD as a co-substrate. Linolenic acid (C18:3) was poorly epoxidised by both isoforms (Figure 5A).

Next the metabolism of fatty acid hydroperoxides in the presence of the purified recombinant proteins PdCLO2 and PdCLO4 was analyzed by HPLC. After 2 h of incubation, PdCLO2 poorly reduced 13-HPOD but more actively reduced 9-HPOD, with about 81 and 16% of the substrates remaining intact respectively (peaks 1 and 2 in Figure 5B, panel I). Conversely, PdCLO4 was more active in the reduction of 13-HPOD rather than 9-HPOD where about 10 and 85% of the substrates remained intact, respectively (peaks 1 and 2 in Figure 5C, panel I). Peak 3 and peak 4 were identified respectively as the alcohol products 13-HOD and 9-HOD, by co-elution with standards (panel II). No metabolites were detected from incubations of 13-HPOD and 9-HPOD with heat-inactivated PdCLO2 and PdCLO4 (panel III). These data indicate that PdCLO2 and PdCLO4 are differentiated by their substrate specificities, with PdCLO2 showing more specificity to reduce 9-HPOD, while PdCLO4 more actively reduced 13-HPOD.

### Reduction of fatty acid hydroperoxides by PdCLO activities is modulated by TCDD and varies according to protein localization

**Figure 6:**
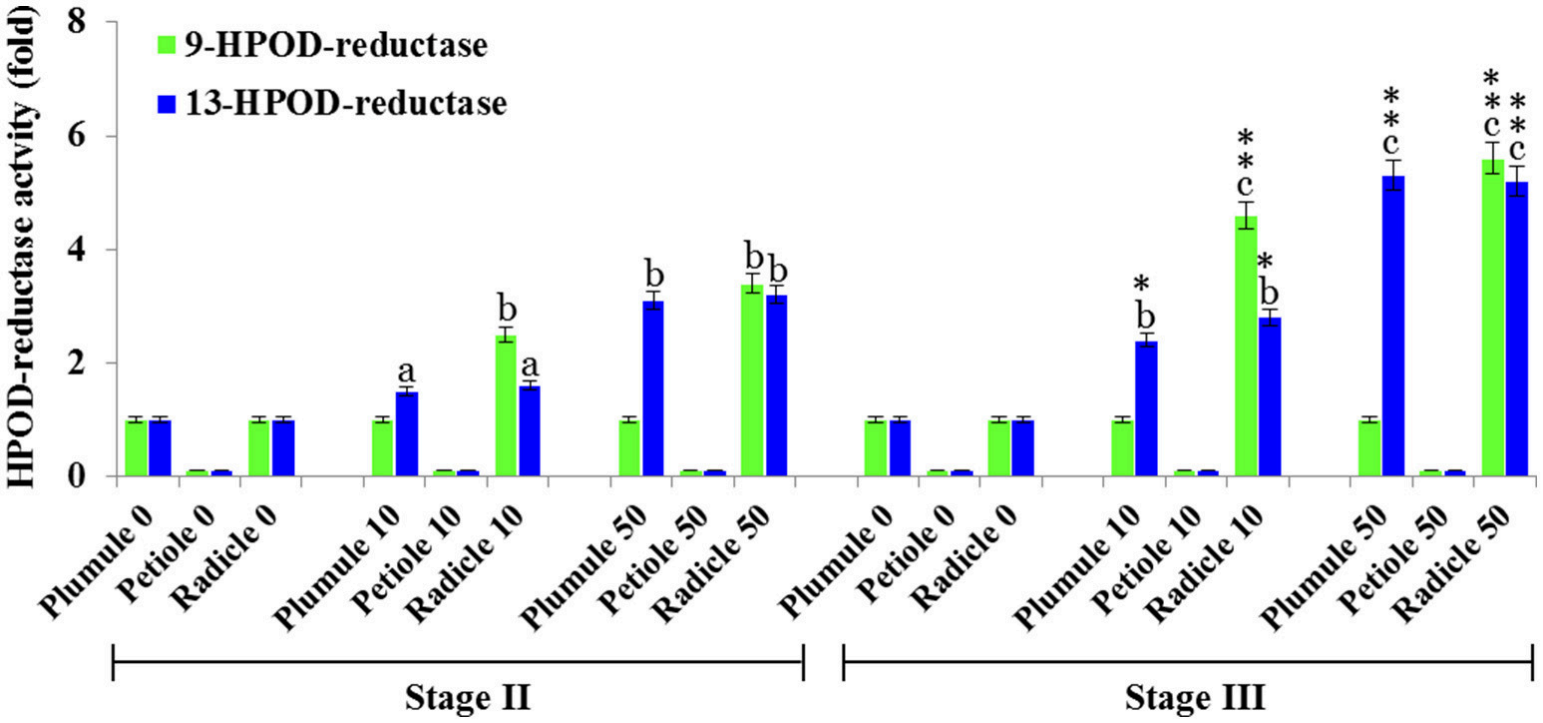
**HPOD-reductase activity is modulated by TCDD**. 13- and 9-HPOD-reductase activities in plumule, petiole, and radicle crude extracts from seedlings at stage II and III were UV-HPLC analyzed as a function of the exposure to various doses of TCDD (0, 10, and 50 ng L^−1^). Fold-change in activity was calculated for each treatment compared with its respective control (non-treated). The measurements were done in triplicate. Values are the means ± S.D. (*n* = 3). Different lowercase letters indicate significant differences (*P* < 0.05) between activities in different tissues for a given stage. Asterisks indicate significant differences between activities in different stages for a given tissue (^*^P < 0.05; ^**^P < 0.01).

The 13- and 9-HPOD-reductase activities in plumule, petiole, and radicle extracts from seedlings in stage II and III were analyzed after exposure to various doses of TCDD. 13-HPOD-reductase activity was significantly increased about 3-fold and more than 5-fold in plumule extract of stage II and III, respectively, as a response to the highest dose of TCDD-exposure (Figure 6). The relatively small 9-HPOD-reductase activity of plumule extracts did not change after TCDD-treatments. Interestingly, the enzymatic activity of radicle extracts in transforming either 13- or 9-HPOD as a function of TCDD treatment increased progressively in stage II and stage III and was maximal in stage III radicle extracts at the highest dose of TCDD (Figure 6). These data show a possible induction of PXG activity in leaves and more particularly in roots of seedlings exposed to TCDD.

### TCDD also induces 9- and 13-LOXs activities by a tissue-specific manner

**Figure 7:**
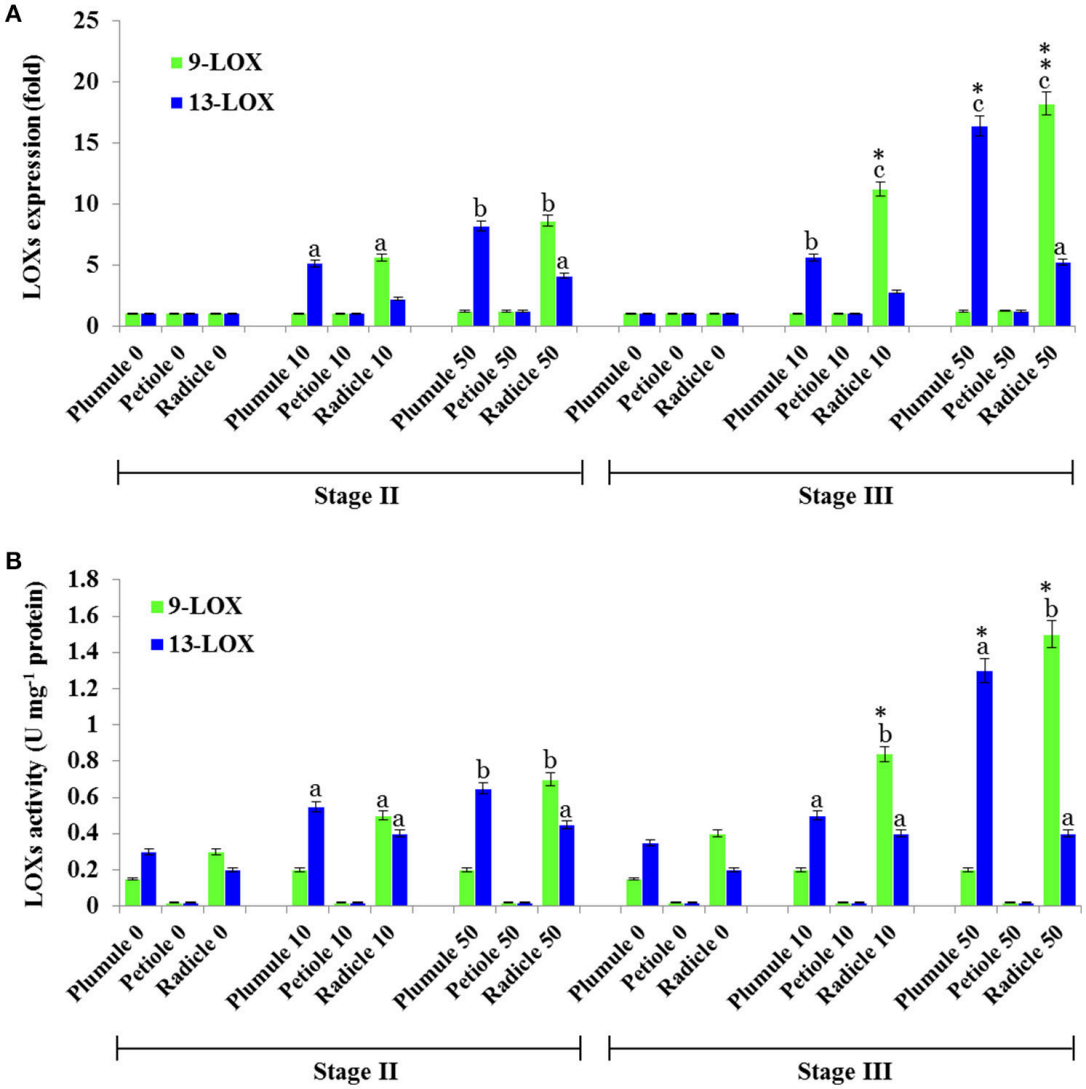
**Profiling of ***9-*** and ***13-LOX-lik***e genes expression and their enzymatic activities in seedling tissues of date palm as a response to TCDD exposure. (A)** Transcriptional analysis of the relative expression levels of 9-LOXs and 13-LOXs encoding genes in various tissues of date palm seedlings in stage II and III after exposure to TCDD (0, 10, and 50 ng L^−1^). For each gene, three measurements were taken in three cDNAs prepared from three individual plants tissues for each treatment. For each gene, the expression levels in seeds and seedlings of date palm unexposed to TCDD were defined as 1, and the corresponding abundance changes under 0, 10, and 50 ng L^−1^ TCDD were calculated directly using the software installed in the Applied Biosystems qPCR system. **(B)** 9- and 13-LOX activities were measured using linoleic acid (C18:2) as a substrate. The reaction products were analyzed by HPLC. Concentrations of products (9- and 13-HPOD) were determined using a standard curve calculated from various known concentrations of 9- and 13-HPOD against the UV peak areas which were recorded at 234 nm by HPLC. Each bar represents the mean and standard error of two replicates. Different lowercase letters indicate significant differences (*P* < 0.05) between 9- and 13-LOX expression or activities in different tissues for a given stage. Asterisks indicate significant differences between 9- and 13-LOX expression or activities in different stages for a given tissue (^*^P < 0.05; ^**^P < 0.01).

In the oxylipin pathway immediately upstream of the PXG activities characterized above are 9-LOX and 13-LOX enzymes that catalyze the hydroperoxidation of unsaturated fatty acids (Feussner et al., 2001). The quantification of transcripts levels for *9-* and *13-LOX-like* genes, (Figure 7) showed that transcripts of *9-LOX-like* genes were specifically increased in root tissues that had been exposed to TCDD. This increase occurred progressively in the roots of both stage II and III and reached its maximum (about 18-flod) in the roots of seedling at stage III after administration of 50 ng L^−1^ TCDD (Figure 7A). While the transcripts of *13-LOX-like* genes also increased in plumule and in roots of both stages as a function of TCDD-exposure, the increase of *13-LOX-like* transcripts was higher in the plumule than in the root (~16-fold vs. ~12-fold). Similar to their transcriptional induction, 9-LOX and 13-LOX activities were significantly and differentially augmented in seedling tissues after treatment with TCDD (Figure 7B). C18:2 was more actively transformed into its corresponding hydroperoxide (9-HPOD) under the action of the root extract and the maximal 9-LOX activity was detected in root extracts from both stages II and III (~ 2.5-fold) compared the root extracts of control plants. In contrast, the activity of 13-LOX in hydroperoxidation of C18:2 was similarly stimulated in the plumule as well as in the radicle of TCDD-treated-seedlings (~2-fold) (Figure 7B). Altogether these data indicate that 9- and 13-LOX activities are induced in a tissue specific manner by TCDD. While 9-LOX activity was uniquely stimulated in roots, similar activation in 13-LOX activity was found in both plumule and roots.

### Bioinformatic analysis of 9- and *13-LOX-like* genes and proteins in date palm and other selected plants

Bioinformatic analysis showed that the genome of date palm harbors nine potential orthologs of *lipoxygenase-like (LOXs-like)* genes, six of which encode putative 13-LOX proteins and three of which encode putative 9-LOX proteins as summarized in Table 3. This Table also shows comparative data from two closely related monocot tree crop plants, i.e., oil palm and banana, for which full genomic sequence data are available, plus the model dicot species, *Arabidopsis thaliana*. In general the 9-LOX proteins are slightly smaller than the 13-LOX proteins with lengths ranging from 847 to 885 and 865 to 926 residues respectively. In the particular case of date palm sequences, the 9-LOX and 13-LOX proteins ranged from 850 to 885 and 896 to 916 residues respectively. With only one exception, all of the 9-LOX sequences lacked a predicted plastid signal peptide as determined by several independent algorithms. The anomalous Arabidopsis sequence, At_9-LOX2 is annotated on the GenomeNet database as a chloroplastic protein

**Table 3:** **Sequence analysis of 9-LOX-like and 13-LOX-like proteins in date palm and other selected plants**.

	**Species Name**	**Sequence Name**	**NCBI Accession**	**Sequence length**	**MW (kDa)**	**Isolectric point (pl)**	**Charge (pH7)**	**13-LOX type**
**13-LOX**	*Phoenix dactylifera*	Pd_13-LOX1	XP_008778427	899	101.77	5.84	−16.81	II
		Pd_13-LOX2	XP_008791914	910	102.53	7.20	1.09	I
		Pd_13-LOX3	XP_008784273	912	102.47	8.14	5.40	I
		Pd_13-LOX4	XP_008782663	916	103.12	7.70	3.24	I
		Pd_13-LOX5	XP_008807550	915	103.06	8.73	10.85	I
		Pd_13-LOX6	XP_008787302	896	102.29	6.67	−2.20	II
	*Elaeis guineensis*	Eg_13-LOX1	XP_010943567	900	101.98	6.17	−9.80	II
		Eg_13-LOX2	XP_010928833	897	101.63	6.14	−10.80	II
		Eg_13-LOX3	XP_010913914	901	102.40	6.53	−3.40	II
		Eg_13-LOX4	XP_010943562	900	101.30	6.55	−4.20	II
		Eg_13-LOX5	XP_010943563	899	101.11	6.55	−4.20	II
		Eg_13-LOX6	XP_010943564	865	97.33	7.14	0.80	II
		Eg_13-LOX7	XP_010943565	797	89.97	6.53	−4.20	II
	*Musa acunimata*	Ma_13-LOX1	XP_009417301	903	101.93	6.31	−8.40	II
		Ma_13-LOX2	XP_009391881	907	101.97	6.37	−7.50	II
	*Arabidopsis thaliana*	At_13-LOX1	AEE77997	896	102.04	5.43	−22.60	II
		At_13-LOX2	AEE29585	919	103.72	7.69	3.20	I
		At_13-LOX3	AEE35334	926	104.81	7.09	0.70	I
		At_13-LOX4	AEE34664	917	104.51	7.89	3.90	I
**9-LOX**	*Phoenix dactylifera*	Pd_9-LOX1	XP_008777614	885	98.69	5.63	−18.23	
		Pd_9-LOX2	XP_008801705	867	98.13	6.02	−11.25	
		Pd_9-LOX3	XP_008791769	850	97.07	7.46	2.17	
	*Elaeis guineensis*	Eg_9-LOX1	XP_010905215	866	98.18	6.26	−7.70	
		Eg_9-LOX2	XP_010935040	851	96.39	5.58	−19.10	
		Eg_9-LOX3	XP_010934565	885	99.21	5.88	−13.10	
		Eg_9-LOX4	XP_010912798	850	97.34	6.69	−2.10	
	*Musa acunimata*	Ma_9-LOX1	XP_009407551	872	98.61	6.03	−15.00	
		Ma_9-LOX2	XP_009387657	861	96.37	6.05	−13.40	
		Ma_9-LOX3	XP_009387658	847	95.18	6.31	−7.70	
		Ma_9-LOX4	XP_009415714	847	95.49	6.33	−7.70	
		Ma_9-LOX5	XP_009406047	873	97.34	6.00	−10.10	
		Ma_9-LOX6	XP_009406046	873	97.33	5.78	−14.10	
	*Arabidopsis thaliana*	At_9-LOX1	NP_175900	859	98.04	5.34	−27.90	
		At_9-LOX2	AEE76630	886	101.06	6.16	−10.50	

*Accession numbers were parsed from NCBI database (http://www.ncbi.nlm.nih.gov). Molecular weights and isoelectric points were calculated using ExPASy (http://expasy.org/). Amino acid net charges were calculated using Bachem (www.bachem.com). 13-LOX sequences were divided into type I and type II based as discussed in the text*.

**Figure 8:**
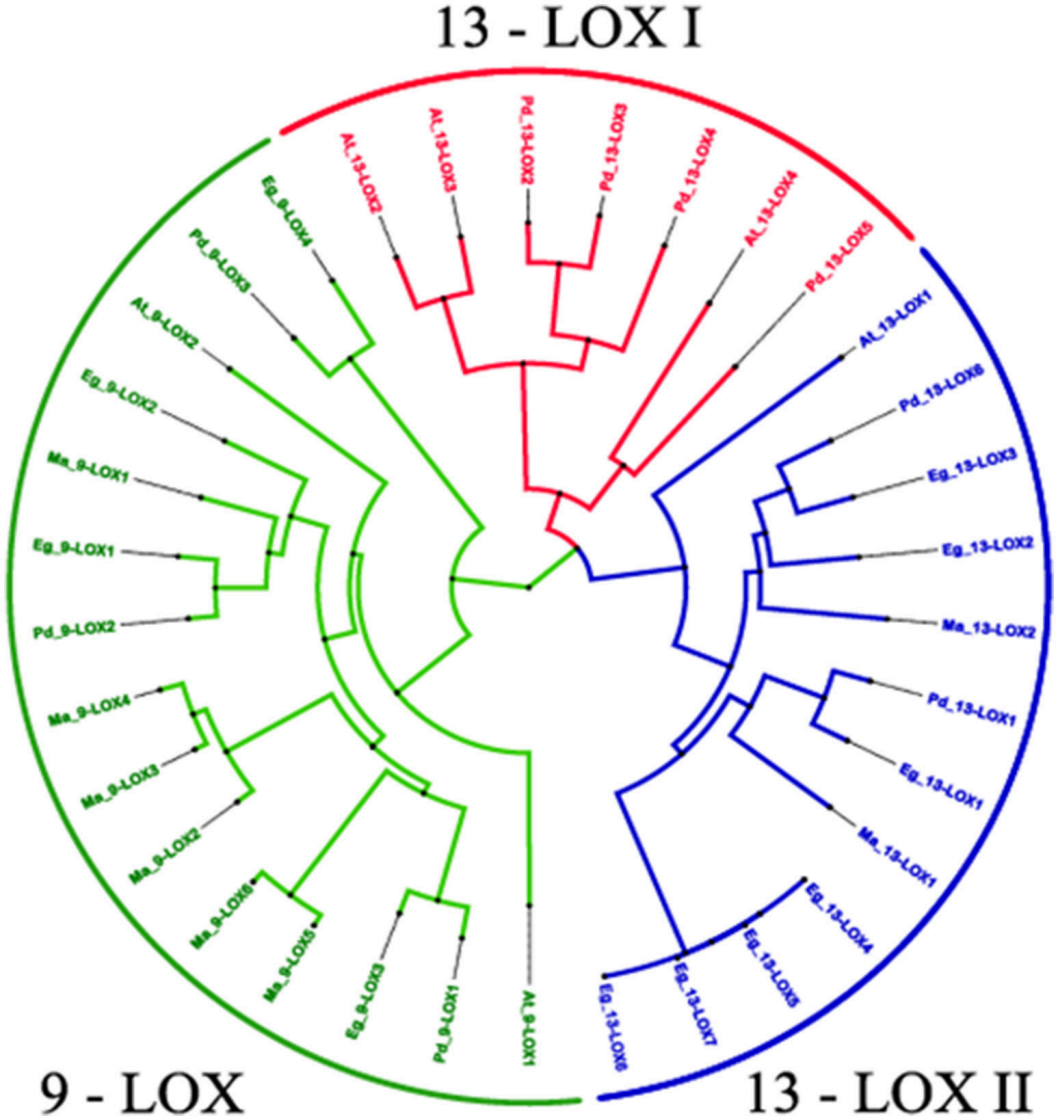
**Phylogenetic relationships between 34 identified 9-LOX and 13- LOX proteins from four ***Phoenix dactylifera***, ***Elaeis guineensis***, ***Musa acuminate*** and ***Arabidopsis thaliana*****. The tree is based on Bayesian analysis and was generated using the neighbor-joining algorithm method of the ClustalW2 program and viewed in FigTree. Branch length is indicated by the scale bars. Taxon labels are indicated in red for the 13-LOX I clade, in blue for the 13-LOX II clade and in green for the 9-LOX clade. See text for further details and discussion.

A phylogenetic analysis of the 38 LOX sequences from the four selected plant species is presented in Figure 8. This clearly shows the clustering of the three LOX groups, namely 9-LOX, 13-LOX type I, and 13-LOX type II with each of the four analyzed plant species having at least one isoform of each of the three LOX groups. Fully aligned sequences of the 15 identified 9-LOX and 19 identified 13-LOX proteins from the four selected plant species are presented in Figures S1, S2. In both the 9-LOX and 13-LOX proteins there are clusters of highly conserved amino acids located throughout these large proteins, which total between 847 and 926 residues in length. In Figures S3, S4 more detailed alignments are shown in the central region that includes the canonical histidine-rich domain that is a feature of all such lipoxygenases. This 38-residue motif has the following structure: [-H-X_4_-H-X_4_-H-X_17_-H-X_8_-H-] and is located at approximately residues 550–590 in 9-LOX and residues 610–650 in 13-LOX sequences. In addition to the five invariant histidines seen in all LOX proteins, well over half of the other residues in this canonical plant 9-LOX and 13-LOX domain were absolutely conserved even between species as divergent as oil palm and Arabidopsis. This indicates that not only the metal- and heme- coordinating histidines have important catalytic and structural functions but also most of the residues depicted above as “X.”

## Discussion

In this study we report the characterisation of two members of the CLO/PXG gene/protein family in date palm (*Phoenix dactylifera* L.) with regard to their tissue localisation, temporal expression, response to TCDD exposure, and the substrate specificities of recombinant proteins expressed in yeast cells. We selected date palm as a candidate for study because it is a perennial monocotyledonous species that is well acclimatized to semi-desert environments where it especially thrives in dry sandy soils. It has also been used as a plant model to study the possibile use of seed LDs as sequestration agents against the toxic pollutant, 2,3,7,8-tetrachlorinated dibenzo-*p*-dioxin (TCDD) (Hanano et al., 2016). Moreover, the complete genome sequence of date palm is publicly available (Al-Mssallem et al., 2013) and our previous transcriptomic data showed that genes encoding caleosins *PdCLO2* and *PdCLO4* were particularly highly expressed in response to TCDD exposure (Hanano et al., 2016). This is the first report of enzymatic activities and specificities of CLO-like proteins in date palm with a particular focus in their regulation in early stages of seedling development and their responses to dioxin exposure. The latter aspect of the study was aimed at providing information on oxylipin-based responses to dioxin toxicity and the potential for the use of plants such as date palm for environmental bioremediation.

As found in seeds from Arabidopsis and oats (*Avena sativa*), our data showed that microsomes and LDs isolated from germinated seeds of date palm were able to catalyze co-oxidation reactions typically known to be mediated by caleosin/peroxygenases (Hanano et al., 2006; Mosblech et al., 2009; Meesapyodsuk and Qiu, 2011; Benaragama, 2015). In quantitative terms, LDs fractioned from an early stage of germination exhibited an order of specific activity comparable to that reported for oat seed LDs (about 40 nmol of epoxide formed min^−1^ mg^−1^ protein) (Hanano et al., 2006), and this activity increased considerably in later stages of seedling growth. As the genome of date palm harbors five putative CLO-like genes, the overall PXG activity estimated in LDs is likely due to the presence of several of these isoforms as also found in Arabidopsis (Hanano et al., 2006; Blée et al., 2012). We have recently reported that two of the five CLO-like genes in the date palm genome, namely *PdCLO2* and *PdCLO4*, were particularly highly expressed during germination and even more after TCDD-treatment (Hanano et al., 2016). We therefore consider that these two CLO isoforms probably contribute the bulk of the measured PXG activities in date palm. Therefore, the corresponding genes were cloned and sequenced to produce recombinant proteins in a heterologous yeast expression system. It should be noted, however, that two other date palm CLO genes, namely *PdCLO1* and *PdCLO3*, were expressed at low levels in seedling tissues and may be expressed at higher levels in other tissues that were not assayed in this study. For example, it is known that some CLO isoforms are specifically upregulated during storage LD accumulation in developing seeds and *PdCLO1* and *PdCLO3* might have roles in this or other physiological processes (Froissard et al., 2009).

Some CLO/PXG proteins appear to have structural roles associated with LDs and can accumulate to relatively high levels in storage organs such as oil-rich seeds (Naested et al., 2000; Frandsen et al., 2001; Hernandez-Pinzon et al., 2001; Murphy et al., 2001). In other cases CLO/PXG proteins have distinctive peroxygenase activities that enable them to generate various long chain fatty acyl oxygenated compounds (Hanano et al., 2006; Blée et al., 2012; Blee et al., 2014). To date, much of the research on the biological functions of the peroxygenases-derived oxylipins has used the plant model *Arabidopsis thaliana* (Blée and Schuber, 1990; Carter et al., 2004; Hanano et al., 2006) and the physiological roles of this family of LD-associated proteins in other plants is much less well known. Our analysis of the CLO amino acid sequences in date palm plus two closely related tree monocot species, i.e., oil palm and banana, and also the model dicot species, Arabidopsis, showed that both of the highly expressed date palm CLO-like proteins contain all of the structural features of plant caleosins previously described (Hanano et al., 2006; Blée et al., 2012).

These characteristics suggested that these two date palm CLO-like proteins can potentially act as peroxygenases. This was confirmed when it was found that the yeast-expressed purified proteins, PdCLO2 and PdCLO4, actively catalyzed peroxygenase (PXG) co-oxidation reactions. However, when cumene hydroperoxide was used as co-substrate PdCLO4 showed more active in epoxidation of FAs than PdCLO2. A similar differentiation was found for AtCLO1 and AtCLO4 from Arabidopsis, where the latter was more active as a FA-epoxidase (Blée et al., 2012). This was confirmed when it was found that PdCLO2 preferentially epoxidised monounsaturated fatty acids (MUFAs) and was less active toward polyunsaturated fatty acids (PUFAs), whereas the inverse case was found for PdCLO4. The specificity of PdCLO4 as a PUFA epoxidase indicates that it is functionally acting similarly to the At CLO3 and AtCLO4 peroxygenases in Arabidopsis (Blée et al., 2012; Blee et al., 2014).

The catalytic differentiation between PdCLO2 and PdCLO4 was further emphasized in regard to the metabolism of fatty acid hydroperoxides (FA-OOHs). Of especial interest, the two studied isoforms differed in their activity toward the most abundant FA-OOHs in plant cells, namely 9-OOH, and 13-OOH, which are formed due to the action of 9-LOX and 13-LOX enzymes respectively (Brash, 1999). Our data demonstrated that while the most active co-substrate for PdCLO2 was 9-HPOD while PdCLO4 preferred 13-HPOD. The ability of these two PXG-isoforms to reduce FA-OOHs is supported by biochemical evidence which was firstly reported for soybean PXG (Blee et al., 1993) and later for the first characterized caleosin in Arabidopsis, AtCLO1 (Hanano et al., 2006). Although the FA-OOHs-reductase activities of AtCLO1 and AtCLO4 were only studied using 13-HPOD or 13-HPOT, respectively (Blée et al., 2012), the specificity of AtCLO3 was evaluated toward both 13-HPOD and 9-HPOD. In line with our results, Blee et al., have recently showed that AtCLO3 metabolizes 13-HPOD more actively rather than 9-HPOD (Blee et al., 2014). Interestingly, we found that PdCLO2 preferentially metabolize 9-HPOD, an unusual activity for the most characterized plant caleosins, raising the question of the physiological roles of this isoform in date palm.

Transcriptomic analysis coupled to enzymatic activity revealed a spatial and temporal shifting in the level of transcripts and activities of both genes *PdCLO2* and *PdCLO4*. The highest expression and 13-HPOD-reductase activity of *PdCLO4* detected in the undifferentiated tissues of leaf at early stage development suggest that *PdCLO4* is more involved in early stages of seed germination, which is in agreement with earlier data published for *AtCLO1* and *AtCLO3* (ATS1 or ATS3) that initially identified as embryo-specific genes in Arabidopsis (Nuccio and Thomas, 1999). In contrast, the highest expression and 9-HPOD-reductase activity of *PdCLO2* in primary roots raises questions about the role of this root-specific caleosin in root development and stress related responses. Interestingly, the differentiation between *PdCLO2* and *PdCOL4* was also shown when transmembrane segments of both proteins were analysed and visualised using transmembrane prediction method (Figure S6).

We carried out bioinformatic analysis of LOX-like genes in the date palm genome, plus the same range of plant species as used for the CLO analysis described above. As depicted in Figures S1, S2, this revealed that the date palm genome contains at least three well conserved *9-LOX-like* genes and six *13-LOX-like* gene sequences that encode putative enzymatic components of two pathways generating 9- or 13-fatty acid hydroperoxides respectively. In all cases these LOX sequences contained the canonical 38-residue histidine-rich motif as found in other plant LOX as depicted in detail Figures S3, S4. Phylogenetic analysis of the 9-LOX and 13-LOX sequences from date palm, oil palm, banana, and Arabidopsis (Figure 8) shows the divergence of the two groups of LOX sequences from the basal level of the tree. As reported for other plant LOX (Chen et al., 2015) there was also a clear separation of type I and type II 13-LOX sequences. In general, type I LOX proteins share high levels of sequences similarity (>75%) and do not contain predicted plastid targeting sequences and are therefore probably located in a cytosolic compartment(s). In contrast, type II LOX proteins have an N-terminal plastid targeting sequence but have lower levels of similarity one to another (Chen et al., 2015). In contrast, the vast majority of 9-LOX proteins are predicted to have cytosolic locations, and this was borne out by the absence of plastid targeting sequences in the species studied here (see Figure S2 and Table 3).

We found a particularly high induction of 9-LOX expression in the roots of TCDD-treated plants (Figure 7). This result is supported by multiple lines of molecular and biochemical evidence that this enzyme has pivotal roles in normal root development. For example, maize *9-LOX* knockout mutants displayed precocious senescence and critically reduced root length (Gao et al., 2008). Likewise, we previously found that TCDD-exposed Arabidopsis exhibited a high level of LOX in its lateral root system (Hanano et al., 2014a, 2015b). Moreover, Vellosillo et al. reported that mutants with defective 9-LOX activity showed increased numbers of lateral roots, suggesting that 9-HOT, or a closely related 9-LOX product, is an endogenous modulator of lateral root formation (Vellosillo et al., 2007). It was also reported that 9-LOX regulates the response to the lipid peroxidation-inducer singlet oxygen suggesting a crucial role for the 9-LOX pathway in modulating oxidative stress, lipid peroxidation, and plant defense (López et al., 2011; Constantino et al., 2013). Beside its involvement in various biological roles such as in the developmental transition to flowering in Arabidopsis (Bañuelos et al., 2009) and storage lipid degradation (Feussner et al., 2001), 13-LOX is also activated in response to a range of biotic and abiotic stresses (Feussner and Wasternack, 2002).

In line with our results regarding the activation of 13-LOX, it was reported that the enhancement of lipoxygenase activity showed to be involved in barley root tip swelling induced by cadmium, auxin or hydrogen peroxide (Alemayehu et al., 2013). Very recently, it was shown that 13-LOX has a key role in chloroplast breakdown as part of programmed cell death in senescing plants (Springera et al., 2016). The 13-LOX enzyme identified in that study accumulated in the plastid envelope and catalyzed the dioxygenation of unsaturated fatty acids of envelope and thylakoid lipids, leading to a selective destruction of the membranes and the release of stromal constituents.

## Conclusions

We have characterized two caleosins isoforms, PdCLO2 and PdCLO4, with peroxygenase activities in seedlings of date palm. These two caleosins are specifically expressed at early stages of seedling development and are highly induced by exposure to the potently toxic dioxin, TCDD. Tissue localization revealed that PdCLO2 has a mainly root-specific PXG activity and preferentially mainly reduces 9-FAOOH, while PdCLO4 is mainly leaf-specific and preferentially reduces 13-FAOOH. Intriguingly, the induction of each caleosin isoform by TCDD coincided with the induction of their respective FA-OOH-forming enzymes, 9-LOX and 13-LOX, suggesting the establishment of two distinct “signatures” of oxylipin metabolism in roots and in leaves as parallel responses to TCDD-exposure. These data highlight the involvement of lipoxygenase/peroxygenase activities as part of a wider oxylipin pathway as a component of the physiological responses that enable date palm plants to tolerate persistent organic pollutants. This supports efforts to establish the use of plant LDs for phytoremediation of environments subject to contamination by such toxins.

## Author contributions

AH led the work, designed all experiments, and co-wrote the manuscript. IA and MS carried out all experimental work. FR and MH performed the bioinformatics analysis. DM designed the computational analysis and co-wrote the manuscript. All authors read and approved the final manuscript.

### Conflict of interest statement

The authors declare that the research was conducted in the absence of any commercial or financial relationships that could be construed as a potential conflict of interest.

## Abbreviations

LDs: lipid droplets
CLO: caleosin
LOX: lipoxygenase
TCDD: 2,3,7,8-polychlorinateddibenzo-p-dioxins
PCDDs: Polychlorinated dibenzo-*p*-dioxins
PCDFs: Polychlorinated dibenzofurans
POPs: persistent organic pollutants.
