# Supplementary material for: Specific Caleosin/Peroxygenase and Lipoxygenase Activities Are Tissue-Differentially Expressed in Date Palm (Phoenix dactylifera L.) Seedlings and Are Further Induced Following Exposure to the Toxin 2,3,7,8-tetrachlorodibenzo-p-dioxin
Source: Front Plant Sci. 2017 Jan 6;7:2025. doi: 10.3389/fpls.2016.02025 (PMC5216026; doi:10.3389/fpls.2016.02025)

Supplementary Information

Legends

Table S1. Name and nucleotide sequences of primers used in this study.

Nucleotides in bold indicate *SacI* and *EcoRI* restriction sites, respectively. Poly-His (6 × CAC) codons were inserted in the N-terminal of forward primers designed for each gene.

Figure S1. 9-LOX multiple sequence alignment of *Phoenix dactylifera, Elaeis guineensis, Musa acuminate* and *Arabidopsis thaliana*

Clustal Omega (clustalo) from ebi.ac.uk was used to align the sequences. CLC sequence viewer from CLCBio was used to visualize the alignments. The multiple sequence alignment residues are coloured according to Rasmol amino colour scheme. The canonical histidine-rich motif is located between residues 553 and 590. The location of the histidine-rich (H) motif is indicated by a red box.

Figure S2. 13-LOX multiple sequence alignment of *Phoenix dactylifera, Elaeis guineensis, Musa acuminate and Arabidopsis thaliana*

Clustal Omega (clustalo) from ebi.ac.uk was used to align the sequences. CLC sequence viewer from CLCBio was used to visualize the alignments. The multiple sequence alignment residues are coloured according to Rasmol amino colour scheme. The canonical histidine-rich motif is located between residues 613 and 650. The location of the histidine-rich (H) motif is indicated by a red box.

Figure S3. Multiple alignment of 9-LOX Histidine-rich motif in *Phoenix dactylifera, Elaeis guineensis, Musa acuminate and Arabidopsis thaliana*

(A) The histidine motif is 38-residues long for each species. The top line shows the conserved histidines (H) for the 15 identified 9-LOX sequences. **(B)** The motif logo was created using MEME software for the identified 9-LOX sequences. The sequence conservation is shown by the overall height of each stack. The height of each residue letter shows the relative frequency of corresponding amino acid residue.

Figure S4. Multiple alignment of 13-LOX Histidine-rich motif in *Phoenix dactylifera, Elaeis guineensis, Musa acuminate and Arabidopsis thaliana*

(A) The histidine motif is 38-residues long for each species. The top line shows the conserved histidines (H) for the 19 identified 13-LOX sequences. **(B)** The motif logo was created using MEME software for the identified 13-LOX sequences. The sequence conservation is shown by the overall height of each stack. The height of each residue letter shows the relative frequency of corresponding amino acid residue.

Figure S5. A simplified workflow of the analysis

The work flow shows steps completed to obtain results.

Supplementary Information

Table S1. Name and nucleotide sequences of primers used in this study.

Target gene	Order Locus	Forward/Reverse Primers (5'-3')	Amplicon (bp)
PdCLO2	LOC103696186	**GAGCTC**ATGCACCACCACCACCACCA<br>CATGGGCCTACCCTACTTCTCT<br>**GAATTC**TCATGCTTTCTTTTGAGAAG	720
PdCLO2q	LOC103696186	GGCGTCCTCATCGTTACCTTGTTCCGG<br>TCAAAGAAGGCGA	106
PdCLO4	LOC103715420	**GAGCTC**ATGCACCACCACCACCACCA<br>CATGACGGACGGCAAGGATGCT<br>**GAATTC**TTAGGATCTCTTATCATTCGA	711
PdCLO4q	LOC103715420	GTCCCTCCCTAAGCCCTACATCCCAAG<br>GGTACACAATGCC	156
Universal 9-LOX	**-**	GACCGTATCTTCTTTGCCAATACGCCG<br>AGGGTAAGGGTACTC	198
Universal 13-LOX	-	CATTGGTTAAGAACTCATGCTCCGGA<br>TCAGGTCAGCTGGCAG	204
TIP-41	AT4G34270	GAACTGGCTGACAATGGAGTG<br>ATCAACTCTCAGCCAAAATCG	96

Figure S1. 9-LOX multiple sequence alignment of *Phoenix dactylifera*, *Elaeis guineensis*, *Musa acuminate* and *Arabidopsis thaliana*

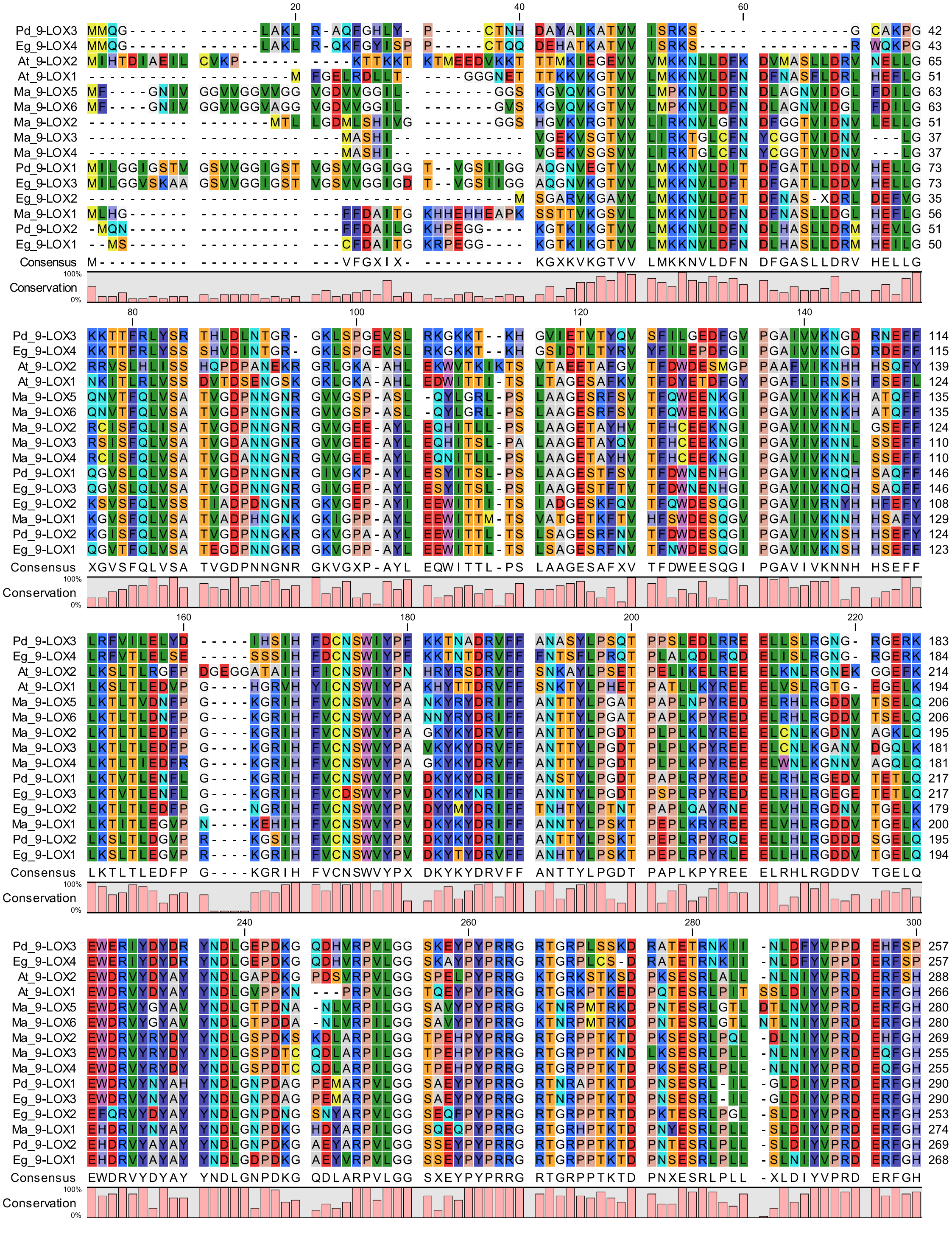

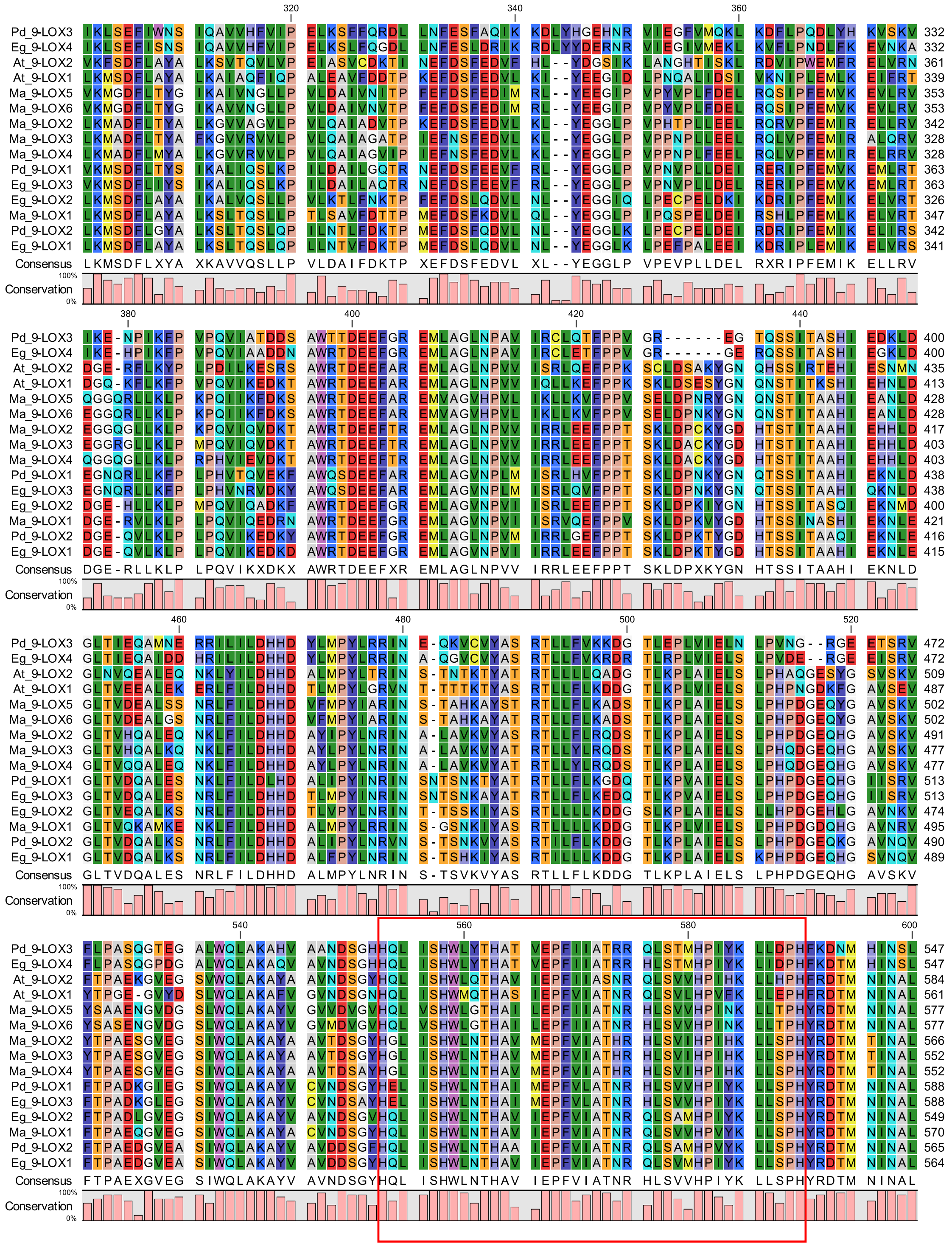

Figure S1 - Page 2

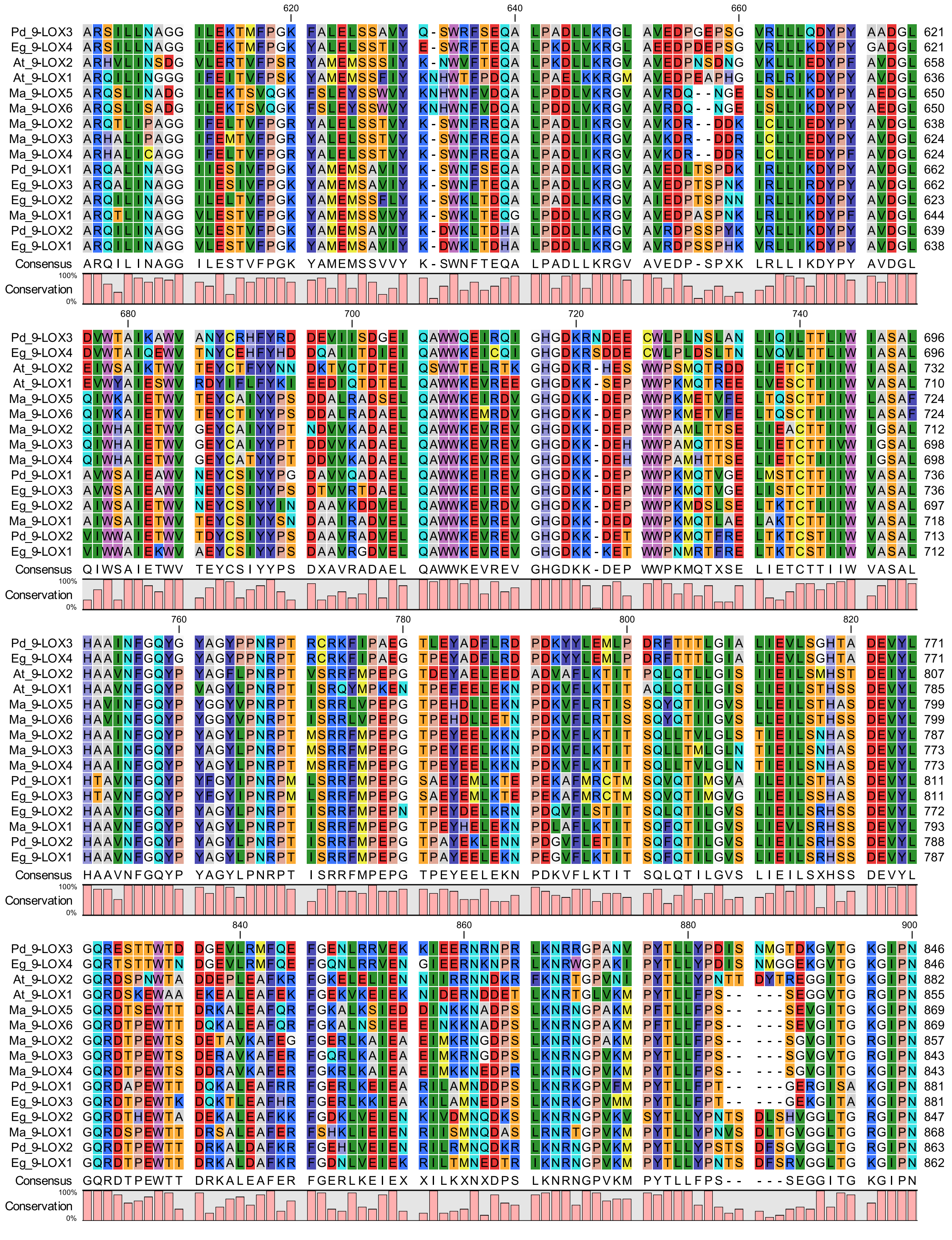

Figure S1 - Page 3

Pd_9-LOX3	S	V	S	I	850
Eg_9-LOX4	S	V	S	I	850
At_9-LOX2	S	V	S	I	886
At_9-LOX1	S	V	S	I	859
Ma_9-LOX5	S	V	S	I	873
Ma_9-LOX6	S	V	S	I	873
Ma_9-LOX2	S	I	S	I	861
Ma_9-LOX3	S	I	S	I	847
Ma_9-LOX4	S	I	S	I	847
Pd_9-LOX1	S	I	S	I	885
Eg_9-LOX3	S	I	S	I	885
Eg_9-LOX2	S	V	S	I	851
Ma_9-LOX1	S	V	S	I	872
Pd_9-LOX2	S	V	S	I	867
Eg_9-LOX1	S	V	S	I	866
Consensus	S	V	S	I	

Conservation

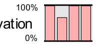

Figure S2. 13-LOX multiple sequence alignment of *Phoenix dactylifera*, *Elaeis guineensis*, *Musa acuminate and Arabidopsis thaliana*

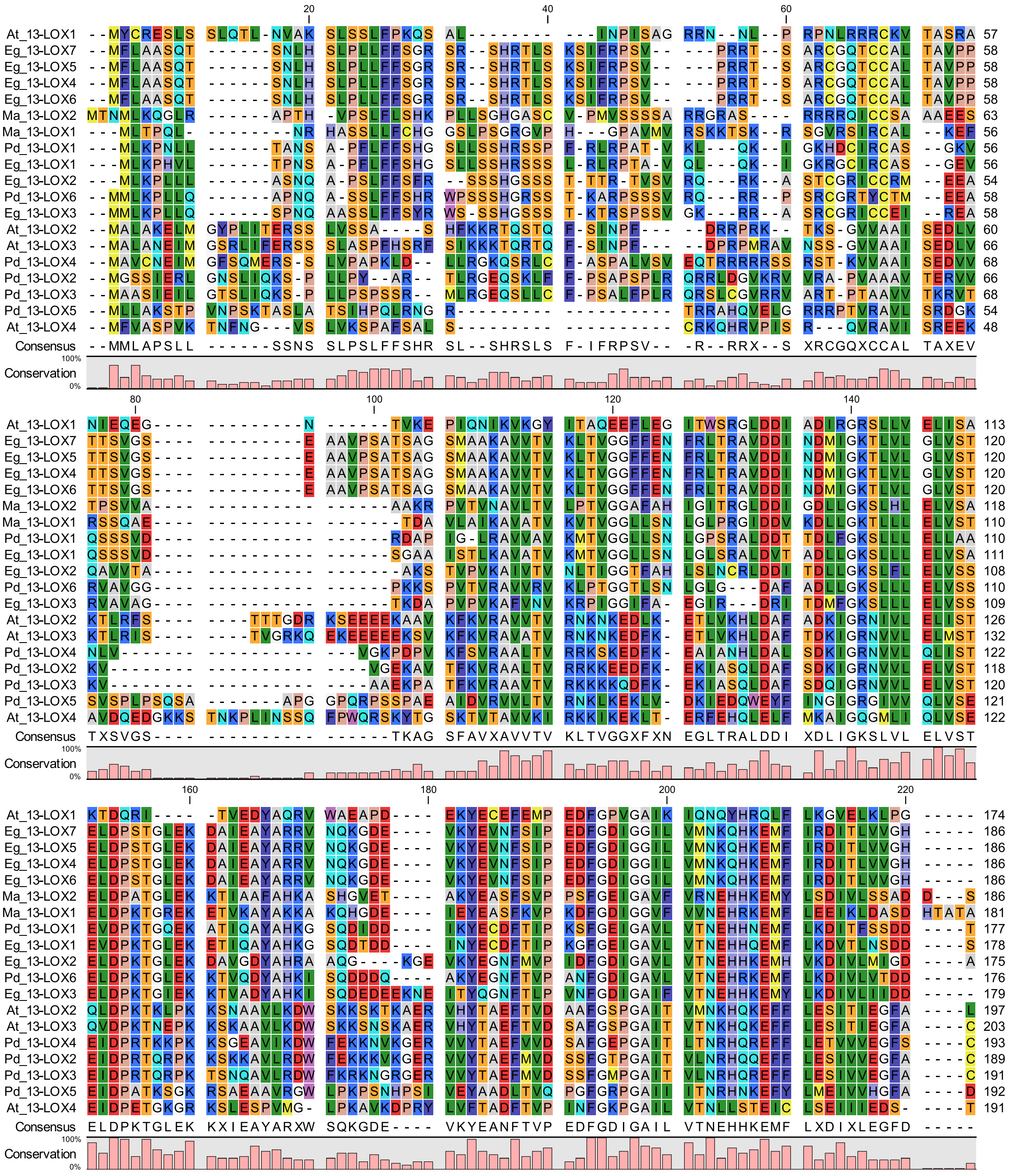

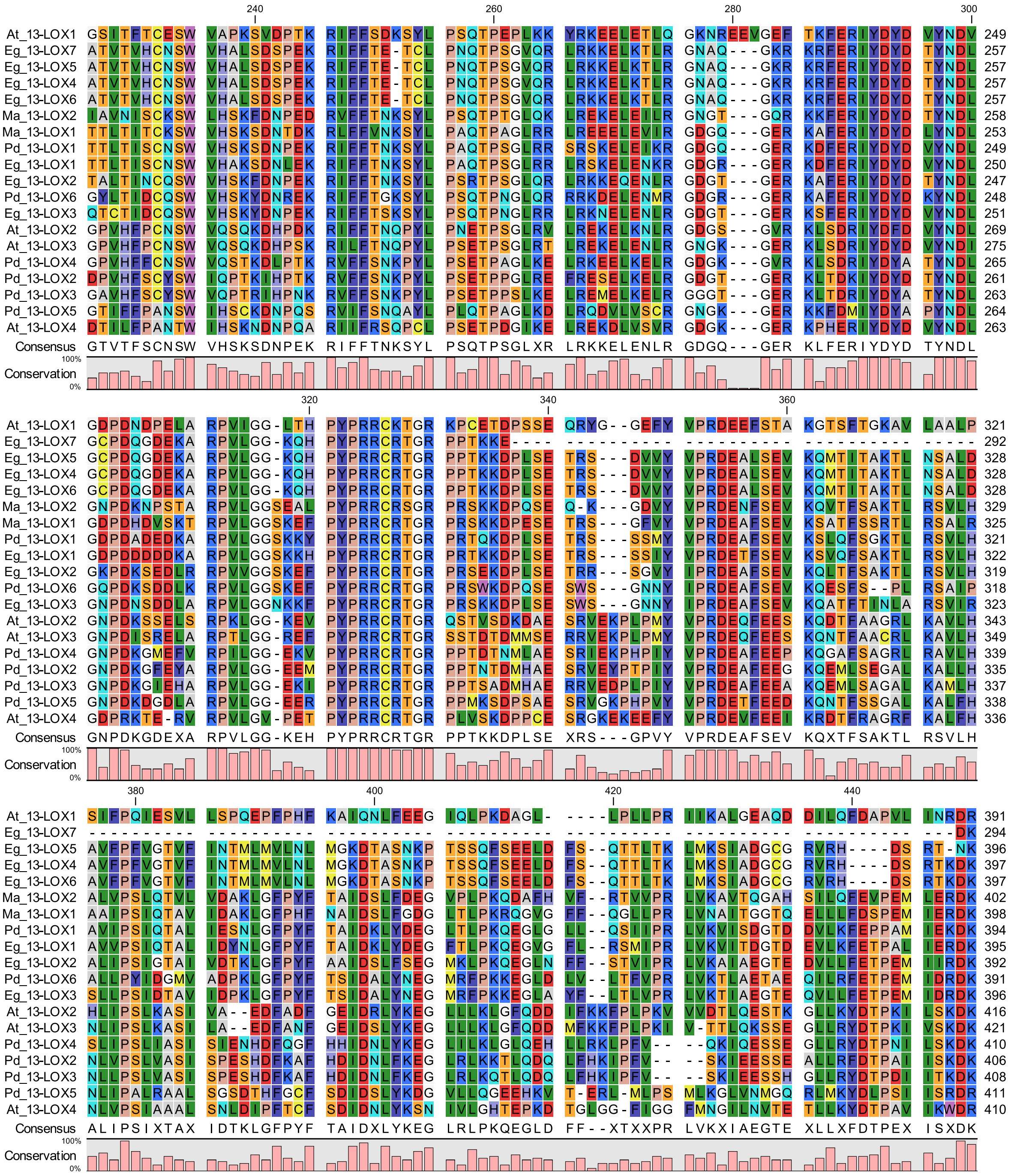

Figure S2 - Page 2

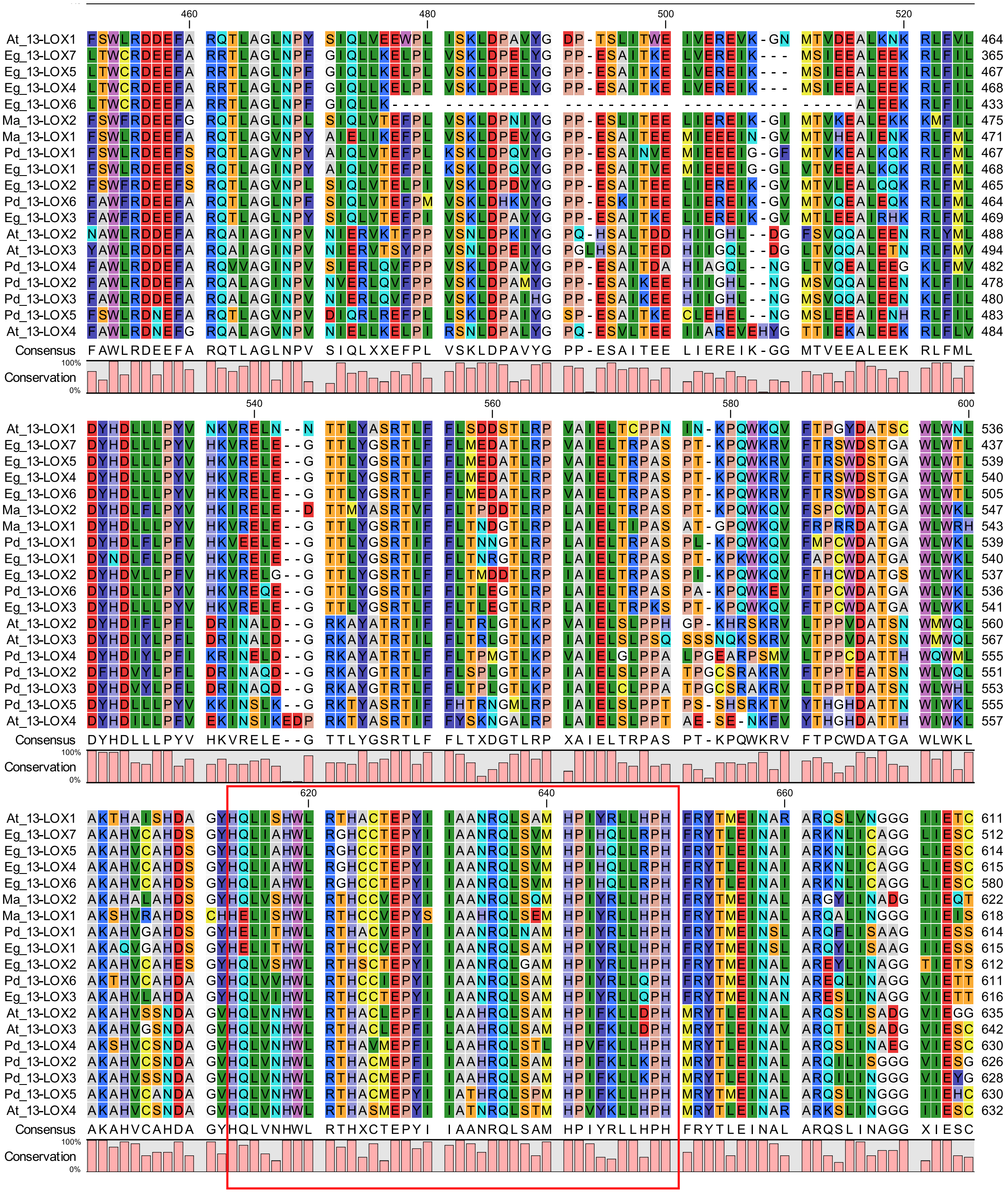

Figure S2 - Page 3

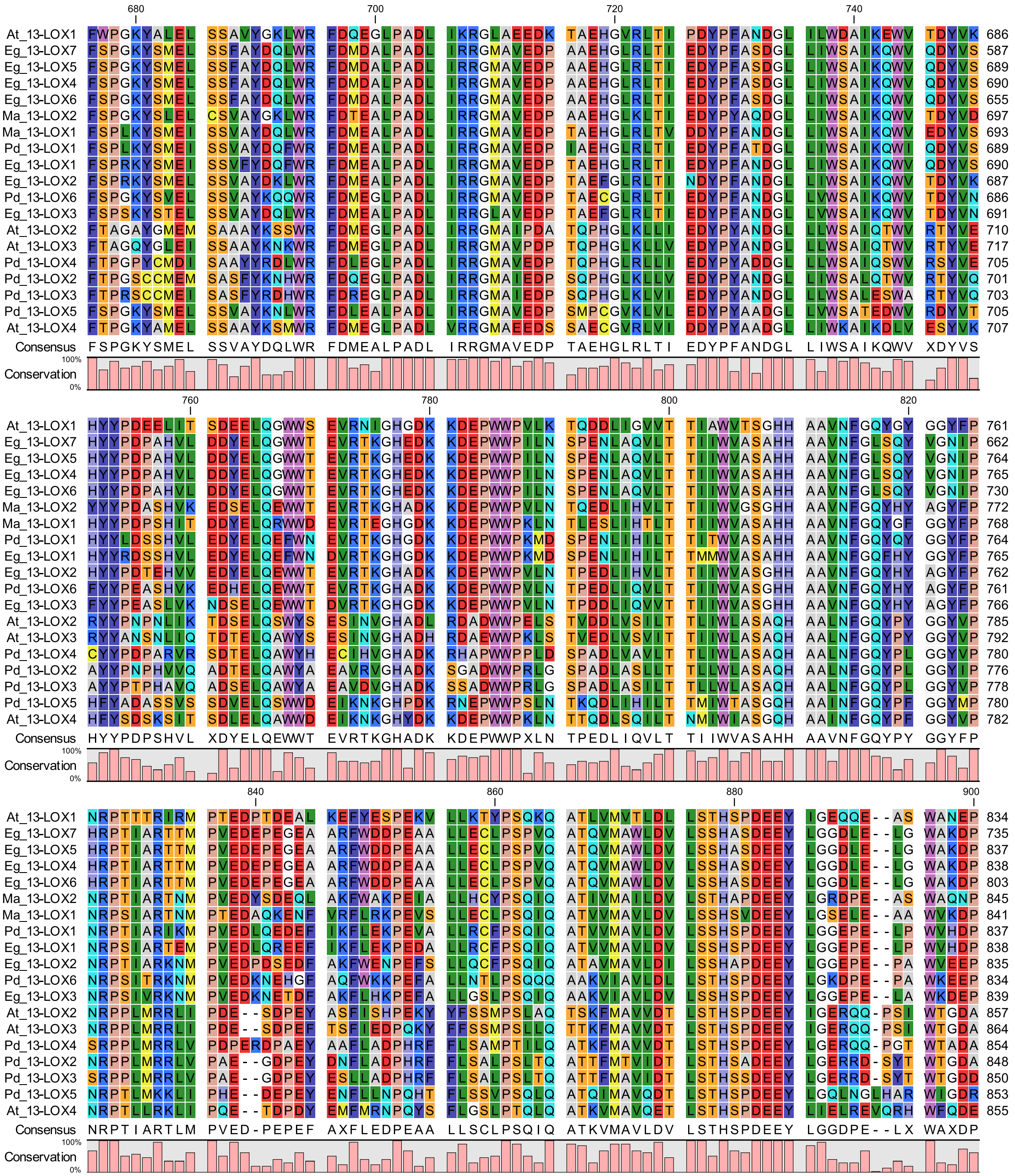

Figure S2 - Page 4

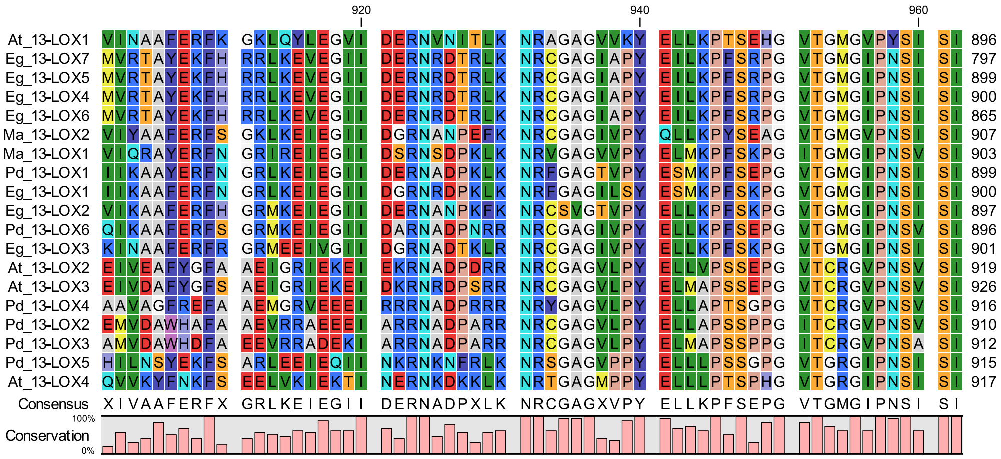
920
940
960
At_13-LOX1 VINAAFERFK GKLQYLEGVI DERNVNITLK NRAGAGVVKY ELLKPTSEHG VTGMGVPYSI SI 896
Eg_13-LOX7 MVRTAYEKFH RRLKEVEGII DERNRDTRLK NRCGAGIAPY EILKPFSRPG VTGMGIPNSI SI 797
Eg_13-LOX5 MVRTAYEKFH RRLKEVEGII DERNRDTRLK NRCGAGIAPY EILKPFSRPG VTGMGIPNSI SI 899
Eg_13-LOX4 MVRTAYEKFH RRLKEVEGII DERNRDTRLK NRCGAGIAPY EILKPFSRPG VTGMGIPNSI SI 900
Eg_13-LOX6 MVRTAYEKFH RRLKEVEGII DERNRDTRLK NRCGAGIAPY EILKPFSRPG VTGMGIPNSI SI 865
Ma_13-LOX2 VIYAAFERFS GKLKEIEGII DGRNANPEFK NRCGAGIVPY QLLKPYSEAG VTGMGVPNSI SI 907
Ma_13-LOX1 VIQRAYERFN GRIREIEGII DSRNSDPKLK NRVGAGVVPY ELMKPFSKPG ITGMGIPNSV SI 903
Pd_13-LOX1 IIKAAYERFN GRLKEIEGII DERNADPKLK NRFGAGTVPY ESMKPFSEPG VTGMGIPNSI SI 899
Eg_13-LOX1 IIKAAFERFN GRLKEIEGII DGRNRDPKLK NRFGAGILSY ESMKPFSKPG VTGMGIPNSI SI 900
Eg_13-LOX2 VIKAAFERFH GRMKEIEGII DERNANPKFK NRCSVGTVPY ELLKPFSKPG VTGMGIPNSI SI 897
Pd_13-LOX6 QIKAAFERFS GRMKEIEGII DARNADPNRR NRCGAGIVPY ELLKPFSEPG VTGMGIPNSV SI 896
Eg_13-LOX3 KINAAFERFR GRMEEIVGII DGRNADTKLR NRCGAGIVPY ELLKPFSKPG VTGMGIPNSI SI 901
At_13-LOX2 EIVEAFYGFA AEIGRIEKEI EKRNADPDRR NRCGAGVLPY ELLVPSSEPG VTCRGVPNSV SI 919
At_13-LOX3 EIVDAFYGFS AEIGRIEKEI DKRNRDPSRR NRCGAGVLPY ELMAPSSEPG VTCRGVPNSV SI 926
Pd_13-LOX4 AAVAGFREFA AEMGRVEEEI RRRNADPRRR NRYGAGVLPY ELLAPTSGPG VTCRGVPNSV SI 916
Pd_13-LOX2 EMVDAWHAFA AEVRRAEEEI ARRNADPARR NRCGAGVLPY ELLAPSSPPG ITCRGVPNSV SI 910
Pd_13-LOX3 AMVDAWHDFA EEVRRADEKI ARRNADPARR NRCGAGVLPY ELMAPSSPPG ITCRGVPNSA SI 912
Pd_13-LOX5 HILNSYEKFS ARLEEIEQII NKRNKNFRLK NRSGAGVPPY ELLLPSSGPG VTGRGIPNSI SI 915
At_13-LOX4 QVVKYFNKFS EELVKIEKTI NERNKDKKLK NRTGAGMPPY ELLLPTSPHG VTGRGIPNSI SI 917
Consensus XIVAAFERFX GRLKEIEGII DERNADPXLK NRCGAGXVPY ELLKPFSEPG VTGMGIPNSI SI
100%
Conservation
0%

Figure S3. Alignment of 9-LOX Histidine-rich motif in *Phoenix dactylifera*, *Elaeis guineensis*, *Musa acuminate* and *Arabidopsis thaliana*

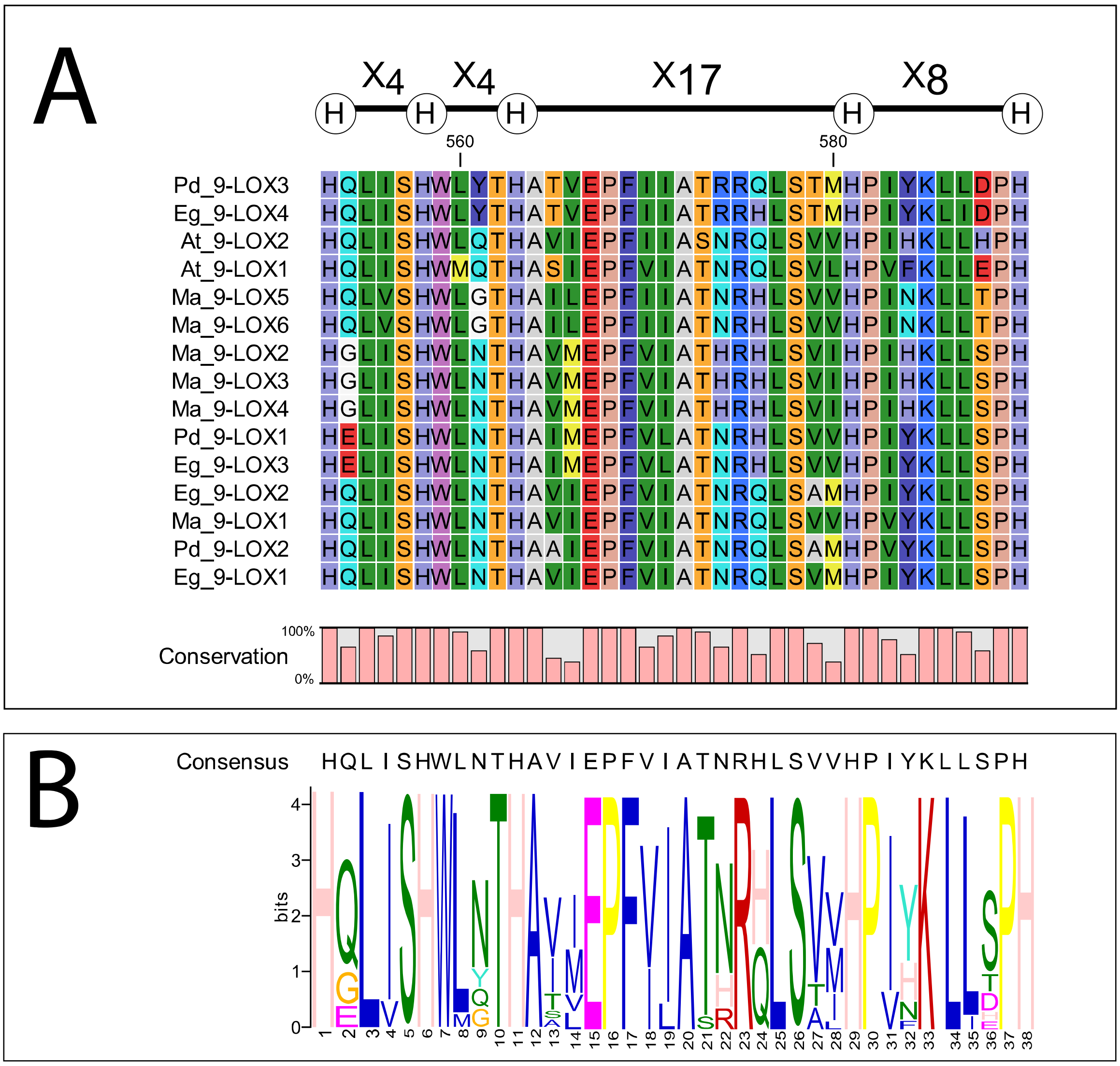

Figure S4. Alignment of 13-LOX Histidine-rich motif in *Phoenix dactylifera*, *Elaeis guineensis*, *Musa acuminate* and *Arabidopsis thaliana*

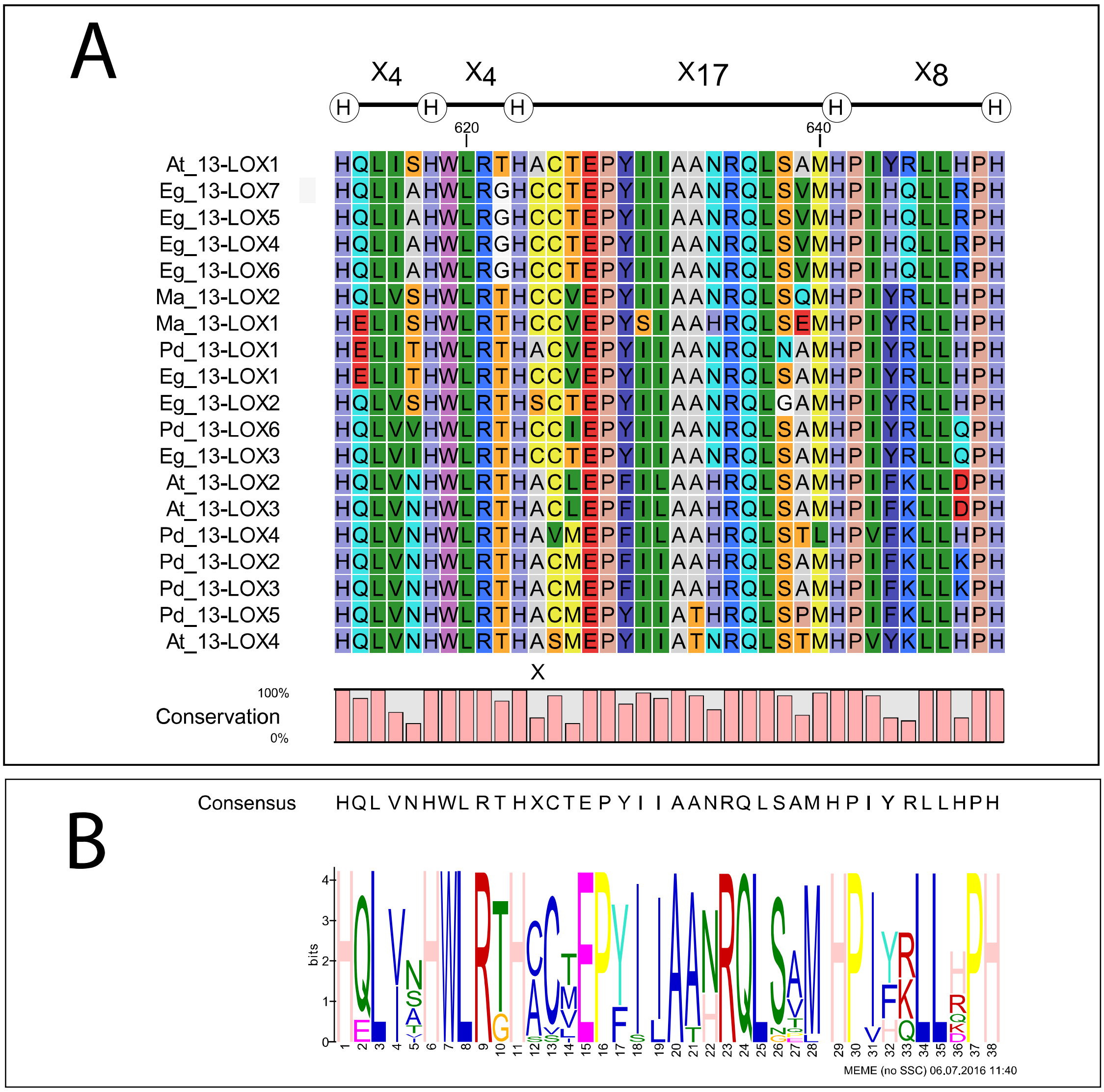

Figure S5. A simplified workflow of computational analysis

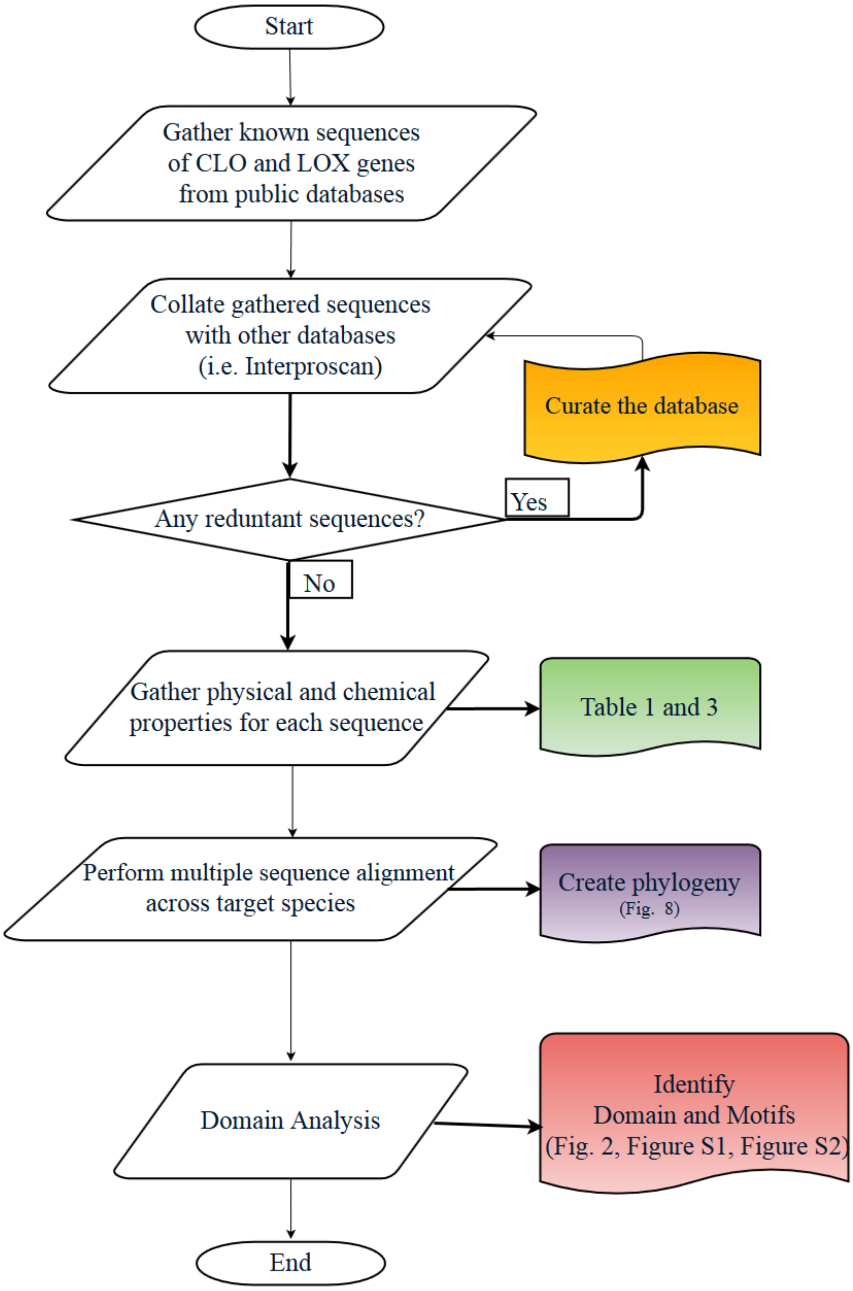

Supplement: Supplementary file 1 [file DataSheet1.PDF]
