# Supplementary material for: Specific Caleosin/Peroxygenase and Lipoxygenase Activities Are Tissue-Differentially Expressed in Date Palm (Phoenix dactylifera L.) Seedlings and Are Further Induced Following Exposure to the Toxin 2,3,7,8-tetrachlorodibenzo-p-dioxin
Source: Front Plant Sci. 2017 Jan 6;7:2025. doi: 10.3389/fpls.2016.02025 (PMC5216026; doi:10.3389/fpls.2016.02025)

Figure S6. Transmembrane prediction using PRED-TMR algorithm

Transmembrane segments were analysed and visualised using transmembrane prediction method of the PRED-TMR algorithm for PdCLO2 **(A)** and PdCLO4 **(B)**. This algorithm is based on a novel statistical analysis method that can predict the location of transmembrane segments using protein sequence information only. The transmembrane prediction algorithm utilises SwissProt database resources.

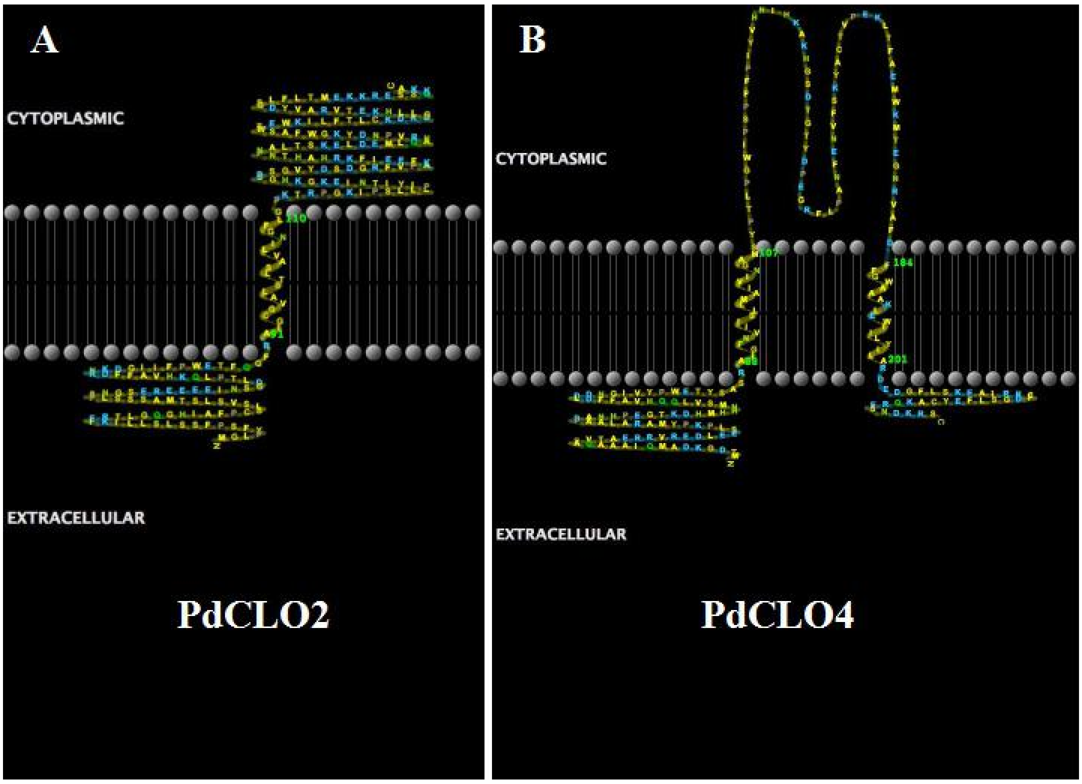

Supplement: Supplementary file 2 [file DataSheet2.PDF]
